# Agronomic Trait Variations and Ploidy Differentiation of Kiwiberries in Northwest China: Implication for Breeding

**DOI:** 10.3389/fpls.2017.00711

**Published:** 2017-05-11

**Authors:** Ying Zhang, Caihong Zhong, Yifei Liu, Qiong Zhang, Xiaorong Sun, Dawei Li

**Affiliations:** ^1^Xian Botanical Garden of Shaanxi Province, Botany Institution of Shaanxi ProvinceXian, China; ^2^Key Laboratory of Plant Germplasm Enhancement and Specialty Agriculture, Wuhan Botanical Garden, Chinese Academy of SciencesWuhan, China; ^3^South China Botanical Garden, Chinese Academy of SciencesGuangzhou, China; ^4^College of Horticulture, Shenyang Agricultural UniversityShenyang, China

**Keywords:** *Actinidia arguta*, kiwiberries, sympatric area, ploidy levels, morphological variation, fruit characters, taxonomy, breeding

## Abstract

Polyploid plants often have higher biomass and superior crop qualities. Breeders therefore search for crop germplasm with higher ploidy levels; however, whether higher ploidy levels are associated with better performance remains unclear. *Actinidia arguta* and related species, whose commercialized fruit are referred to as kiwiberries, harbor a series of ploidy races in nature, offering an opportunity to determine the link between ploidy levels and agronomic traits. In the present study, we determined the ploidy levels of *A. arguta* var. *arguta, A. arguta* var. *giraldii*, and *A. melanandra* in 16 natural populations using flow cytometry, and examined 31 trait variations in fruits, leaves and flowers by field observations, microscopic examination and laboratory analyses. Our results showed that octaploid and decaploid *A. arguta* var. *giraldii* had larger dimension of leaves than tetraploid *A. arguta* var. *arguta* and *A. melanandra*, but their fruits were significantly smaller. In addition, *A. arguta* var. *giraldii* (8*x* and 10*x*) had higher contents of nutrients such as ascorbic acid and amino acids; however, some important agronomic traits, including the content of total sugar and total acid, were significantly lower in the octaploids and decaploids. Moreover, octaploids and decaploids did not result in greater ecological adaptability for the challenging environments and climates. In conclusion, the differentiation of ecological adaptability and traits among natural kiwiberries' cytotypes suggested that higher ploidy levels are not inevitably advantageous in plants. The findings of *A. arguta* and related taxa in geographical distribution and agronomic trait variations will facilitate their germplasm domestication.

## Introduction

Polyploidy, or whole genome duplication, has been an important feature of evolution and diversification in flowering plants (Otto and Whitton, 2000). Recent analysis basing on genomic data inferred all extant angiosperms have descended from polyploid species and undergone one or more chromosomal duplication events (Soltis et al., 2009). Plant polyploidization, is not the sum of parental genotypes (Adams and Wendel, 2005) but rather represents rapid and substantial genome reorganization, gene fractionation, transcriptomic and epigenetic alterations, and sub- and neofunctionalization of duplicate genes (Renny-Byfield and Wendel, 2014). The genomic changes subsequently altered plant physiology, morphology, phenology, and/or ecology within only one or a few generations (Levin, 2002), in particular, improved agronomical traits in some polyploid crops (Dubcovsky and Dvorak, 2007; Leitch and Leitch, 2008).

The connections between ploidy and agronomical traits in crops, however, are more complicated than breeders thought. Polyploidization provide de facto evidence for crop improvement, for example, enhanced cold adaptability in wild tetraploid potatoes (Hijmans et al., 2007) and increased vigor or higher biomass in polyploid cotton (Wendel and Cronn, 2003), rice (Cheng et al., 2007), wheat (Uddin et al., 1992), and maize (Crow, 1998; Duvick, 2001). Other changes in crop quality, such as doubling the amount of soluble proteins in *Panicum virgatum* (Warner et al., 1987), increased amino acid content in sorghum (Luo et al., 1992), improved fruit quality in tomato (Kagan-Zur et al., 1991) and higher secondary metabolite levels in *Cymbopogon* (Lavania et al., 2012), have also been observed. But there are also several disadvantages of polyploidy, including the disrupting effects of nuclear and cell enlargement and the propensity of polyploid mitosis and meiosis to produce aneuploid cells and epigenetic instability, resulting in transgressive (non-additive) gene regulation (Comai, 2005). Similarly, the elevated ploidy level does not consistently increase body size (Lavania, 2013). Even in grain crops, induced autopolyploid significantly increases seed size, but this advantage is offset by the reduction in overall seed set (Dhawan and Lavania, 1996). Basing on the comparison between diploid and its progenies, increasing evidences fuelled speculations that genome duplications may lead to the “dead-ends” (Wagner, 1970). Here, we want to ask more: do organisms with higher ploidy levels or multi-polyploidization (e.g., octoploid or decaploid) really perform better than their ancestors; namely, “the more (chromosome), the better?”

An effective way to evaluate the connections between ploidy level and ecological adaption might be focused on naturally existing that contains a mix of cytotypes. Cytogeographical investigations, particularly in sympatric areas containing different species, have provided valuable information to interpret the ecological adaption and evolutionary patterns (e.g., mating, competition) of different cytotypes (Soltis et al., 2010; King et al., 2012; Sonnleitner et al., 2016). Kiwiberries (sometimes called baby kiwi or hardy kiwifruit), the fruit of *Actinidia. arguta* (Sieb. and Zucc.) Planch. ex Miq. and the related species, *A. melanandra* Franch. and *A. hypoleuca* Nakai, are widely distributed in Asia and particularly diverse in ploidy levels (2*x*, 4*x*, 6*x*, 8*x*, etc.) (Ferguson and Seal, 2008). *Actinidia. arguta* var. *arguta* is distributed throughout eastern Siberia, Korea, Japan, and much of China (Li J. et al., 2007), the closely related *A. hypoleuca* is native to Japan, and *A. arguta* var. *giraldii* (Diels) Vorosh. and *A. melanandra* are unique to China. In Japan, hexaploid and heptaploid *A. arguta* var. *arguta* are found in northern, deep-snow regions and diploid *A. hypoleuca* in warm Pacific hill areas, whereas tetraploid plants of *A. arguta* var. *arguta* are widely distributed throughout Japan (Kataoka et al., 2010; Asakura and Hoshino, 2016), indicating potential ecological sorting among the ploidy races of *A. arguta* var. *arguta* and *A. hypoleuca*. Furthermore, complex ploidy variation (4*x*, 6*x*, 8*x*, and 10*x*) was detected in a population of *A. arguta* var. *arguta* in northwest China (Li et al., 2013). However, studies of other taxa, such as *A. melanandra* and *A. arguta* var. *giraldii*, are lacking. In particular, the ecological adaptation and mechanisms (e.g., niche separation or reproductive isolation, Fowler and Levin, 1984; Van Dijk and Bijlsma, 1994; Suda et al., 2007; Sonnleitner et al., 2016) responsible for the spatial separation or co-existence of these taxa remain far from explicit.

Breeding to take advantage of the diversity of *Actinidia* taxa is a pivotal strategy to broaden the genetic basis of present kiwifruit cultivars (Ferguson, 2007; Ferguson and Huang, 2007; Datson and Ferguson, 2011). The 54 species of *Actinidia* (Li J. et al., 2007; Li X. et al., 2007) characterizing by complex ploidy variation (Ferguson and Huang, 2007), are particularly diverse in fruit characteristics, such as size, shape, skin hairiness, flesh color, flavor, nutrient content, time of maturation, and storage life (Ferguson and Seal, 2008). Kiwiberries appear to be the most promising for further commercialization of kiwifruit (Boyd et al., 2002) because their fruit have edible skins, colorful flesh, good flavor, and functional health components (Matich et al., 2003; Nishiyama et al., 2005, 2008). Except for some elite breeding programmes (Boyd et al., 2002; Bieniek, 2012), there have been few studies concerning the morphological characteristics, fruit quality and sensory analyses of natural resources to explore new fruit characteristics for further breeding. In particular, it is not known whether the biological features, including fruit size, quality, or disease resistance, of some genotypes with higher ploidy levels, such as octaploids and decaploids, might be enhanced as a result of polyploid advantage (Adams and Wendel, 2005; Udall and Wendel, 2006). For example, an autotetraploid of *A. chinensis* var. *chinensis*, derived from chromosome doubling, had significantly larger fruit than its diploid parent (Wu et al., 2012, 2013), and hexaploids [*Actinidia chinensis* var. *deliciosa* (A. Chev.) A. Chev.] are more resistant to *Pseudomonas syringae* pv. *actinidiae* than diploids (*A. chinensis* var. *chinensis*) (Datson et al., 2013).

*Actinidia arguta* and related species, whose have considerable commercial potential, harbor abundant ploidy and morphological variation in nature, providing the opportunity to better understand the relationship between ploidy levels and adaptability or agronomic traits. In this study, we conducted a series of morphometric and cytological investigations on sympatric populations of *A. arguta* var. *arguta, A. arguta* var. *giraldii*, and *A. melanandra* to assess (1) ploidy variation, distribution patterns, and potential co-existence mechanisms; (2) morphological characteristics, fruit quality, and relationships with ploidy levels. Specifically, we discuss the classification and germplasm utilization (e.g., superior germplasm having fruit with higher nutritional advantages or red or purple flesh) of these taxa for future breeding.

## Materials and methods

### Study site and sample investigation

**Figure 1:**
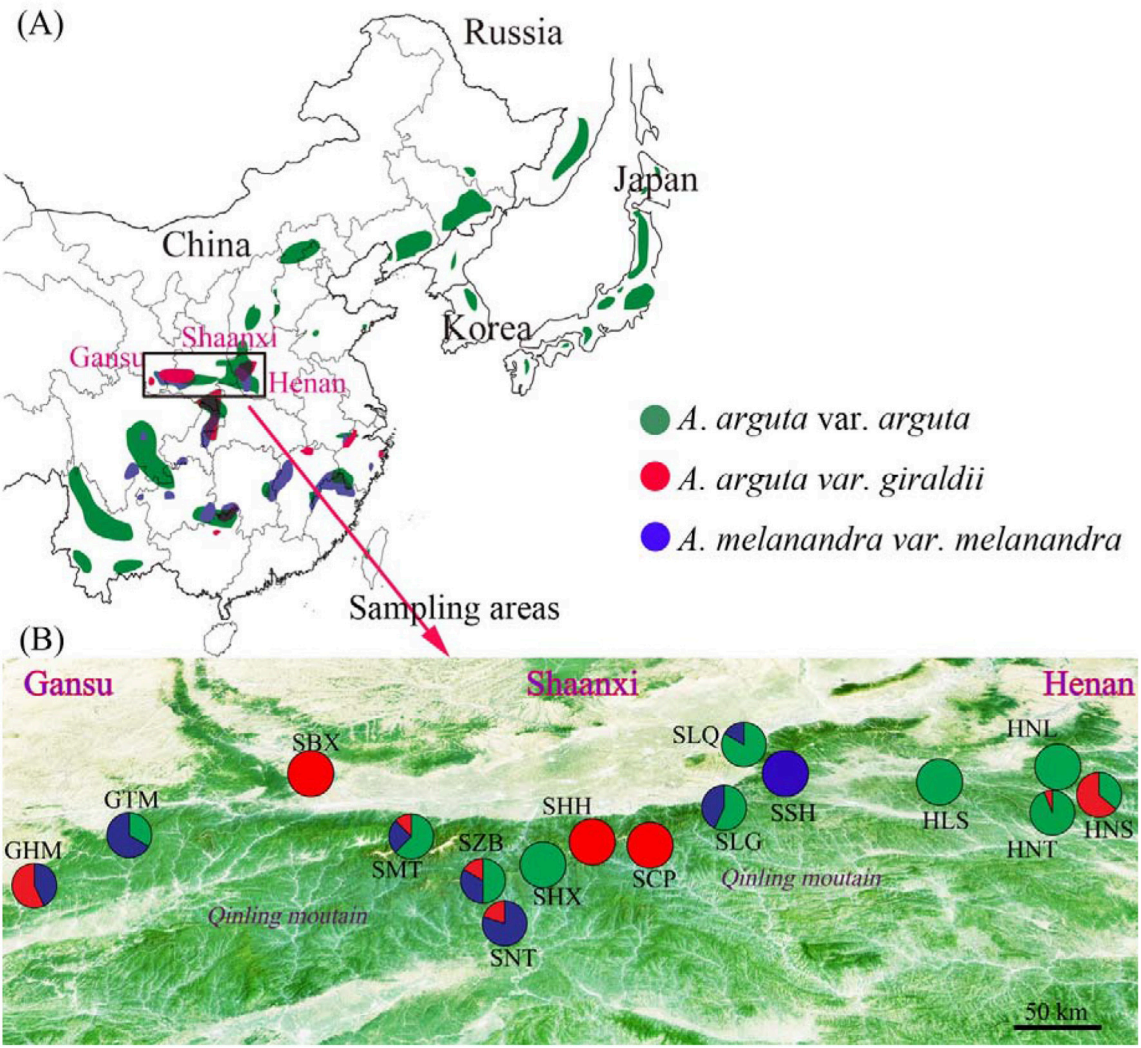
**(A)** Overall geographic distribution of *Actinidia arguta* var. *arguta* (green spots), *A. arguta* var. *giraldii* (red spots), and *A. melanandra* (blue spots), and **(B)** the sympatric areas of three taxa sampled in the present study. The pie diagrams represent the proportion of *A. arguta* var. *arguta* (Green), *A. arguta* var. *giraldii* (red), and *A. melanandra* (blue) in each population. Population names and locations according to Table 1.

The Qinling Mountain in China is the main sympatric areas of natural kiwiberries populations, including *A. arguta* var. *arguta, A. arguta* var. *giraldii*, and *A. melanandra* (Figure 1A). From 2010 to 2015, we collected 16 natural populations across a 700-km region of the Qinling Mountain (33°21′18.42″–34°40′2.9844.138 N and 105°44′6.32″–111°41′46.86″ E; Figure 1B, Table 1). In total, 119 plants with 64 *A. arguta* var. *arguta*, 28 *A. arguta* var. *giraldii*, and 27 *A. melanandra* were systematically investigated over the 5-year period. To better understand the ecological adaptability of kiwiberries, the climate and environmental data were collected and analyzed from each sample site. The altitude, latitude and longitude were recorded by GPS; and the climate data (30 years, 1980–2010) were obtained from China Meteorological Data Service Centre (http://data.cma.cn/) or Local Meteorological Bureau. Here, we calculate the mean value of 30 years climate data as follows: annual cumulative sunshine hours, extreme maximum and minimum temperature, and monthly maximum and minimum temperature, humidity and precipitation.

**Table 1:** **Ploidy and geographical distribution of *Actinidia arguta* var. *arguta, A. arguta* var. *giraldii* and *A. melanandra* in 16 populations from the Qinling Mountain, China**.

**Location**	**Population code**	**Taxon range (m)**	**Longitude and Latitude**	**Altitude**	**Number of ploidy races**		
					**4*x***	**8*x***	**10*x***
Laojielin, Luanchuan County, Henan Province	HNS	*A. arguta* var. *arguta*	111°41′46.86″ E, 33°41′45.02″ N	1148–1152	4		
		*A. arguta* var. *giraldii*		1276–1512		7	
Taiping, Luanchuan County, Henan Province	HNT	*A. arguta* var. *arguta*	111°39′30.51″ E, 33°38′37.99″ N	1005–1431	16		
		*A. arguta* var. *giraldii*		1005		1	
Laojunshan, Luanchuan County, Henan Province	HNL	*A. arguta* var. *arguta*	111°38′12.24″ E, 33°45′04.02″ N	835–1111	13		
Shiziping, Lushi County, Henan Province	HLS	*A. arguta* var. *arguta*	110°51′10.89″ E, 33°47′36.78″ N	1140–1359	7		
Heilongkou, Shangluo County, Shaanxi Province	SSH	*A. melanandra*	109°38′59.01″ E, 34°04′36.01″ N	1371–1378	7		
Qingyu, Lantian County, Shaanxi Province	SLQ	*A. arguta* var. *arguta*	109°35'17.39″ E, 34°13′34.48″ N	1185–1390	5		
		*A. melanandra*		1200	1		
Gepai, Lantian County, Shaanxi Province	SLG	*A. arguta* var. *arguta*	109°28′50.02″ E, 33°53′09.01″ N	1406–1410	8		
		*A. melanandra*		1406–1410	6		
Peiyu, Changan County, Shaanxi Province	SCP	*A. arguta* var. *giraldii*	108°49′56.11″ E, 33°52′38.03″ N	1545–1587		3	
Hegou, Hu County, Shaanxi Province	SHH	*A. arguta* var. *giraldii*	108°26′54.45″ E, 33°52′11.26″ N	1215–1429		4	1
Xiliu, Hu County, Shaanxi Province	SHX	*A. arguta* var. *arguta*	108°23′33.56″ E, 33°48′34.11″ N	1500–1672	2		
		*A. melanandra*		1500–1550	2		
		*A. arguta* var. *giraldii*		1550		1	
Tongche, Nongshan County, Shaanxi Province	SNT	*A. arguta* var. *arguta*	108°02′12.51″ E, 33°21′18.42″ N	601–696	3		
Bangfang, Zhouzhi County, Shaanxi Province	SZB	*A. melanandra*	107°58′30.10″ E, 33°46′03.67″ N	1424–1701	4		
		*A. arguta* var. *giraldii*		1385			1
Taibai, Mei County, Shaanxi Province	SMT	*A. arguta* var. *arguta*	107°42′36.03″ E, 34°05′06.47″ N	1260–1300	5		
		*A. melanandra*		1260–1300	2		
		*A. arguta* var. *giraldii*		1285			1
Xigou, Baoji County, Shaanxi Province	SBX	*A. arguta* var. *giraldii*	107°04′28.71″ E, 34°40′02.98″ N	1232–1350		1	4
Maiji, Tianshui County, Gansu Province	GTM	*A. arguta* var. *arguta*	106°06'48.05″ E, 34°22′45.52″ N	1603	1		
		*A. melanandra*		1560–1606	2		
Mayanhe, Hui County, Gansu Province	GHM	*A. melanandra*	105°44′06.32″ E, 34°05′29.13″ N	1400–1520	3		
		*A. arguta* var. *giraldii*		1455–1481			4
Total: 119 samples					91	17	11

Kiwiberries plants were initially classified as *A. arguta* var. *arguta, A. arguta* var. *giraldii*, or *A. melanandra* following the taxonomic treatment of *Actinidia* (Li J. et al., 2007), and their phenotypic traits were preliminary recorded. In addition, the fruits were harvested and stored fresh in a refrigerator for trait assessment in Northwest A&F University, Yangling, China. Voucher specimens of the leaves were made and deposited in the Herbarium of Xian Botanical Garden (XBG) for microscopic examination using an Olympus BH-2 microscope coupled with a Nikon D800 camera (Tokyo, Japan). The dormant canes were pruned to graft onto rootstocks at the kiwifruit orchard at XBG for phenological investigations, and to sprout new leaves for ploidy analysis at Wuhan Botanical Garden.

### Ploidy examination

The ploidy level of each individual was determined by the flow cytometric measurement (FCM) using a flow cytometry system (Partec Cyflow Space, Germany). The new leaves of each sample were chopped and lysed in nuclear extraction buffer (solution A of High Resolution Kit, Partec, Germany) to extract the cells, followed by chromosome staining of 6-diamidino-2-phenylindole. FCM was based on a linear relationship between the fluorescence signals of the unknown sample and known internal standards. In the present study, individual DNA ploidy levels were calculated after comparing the position of the fluorescence peak of the unknown sample and the internal standard *Actinidia. chinensis* var. *chinensis* “Hongyang” (2*n* = 2*x* = 58), whose chromosome number had previously been determined by counting. The detail experimental procedure followed the basic protocol of Li et al. (2010).

### Trait assessment

**Figure 2:**
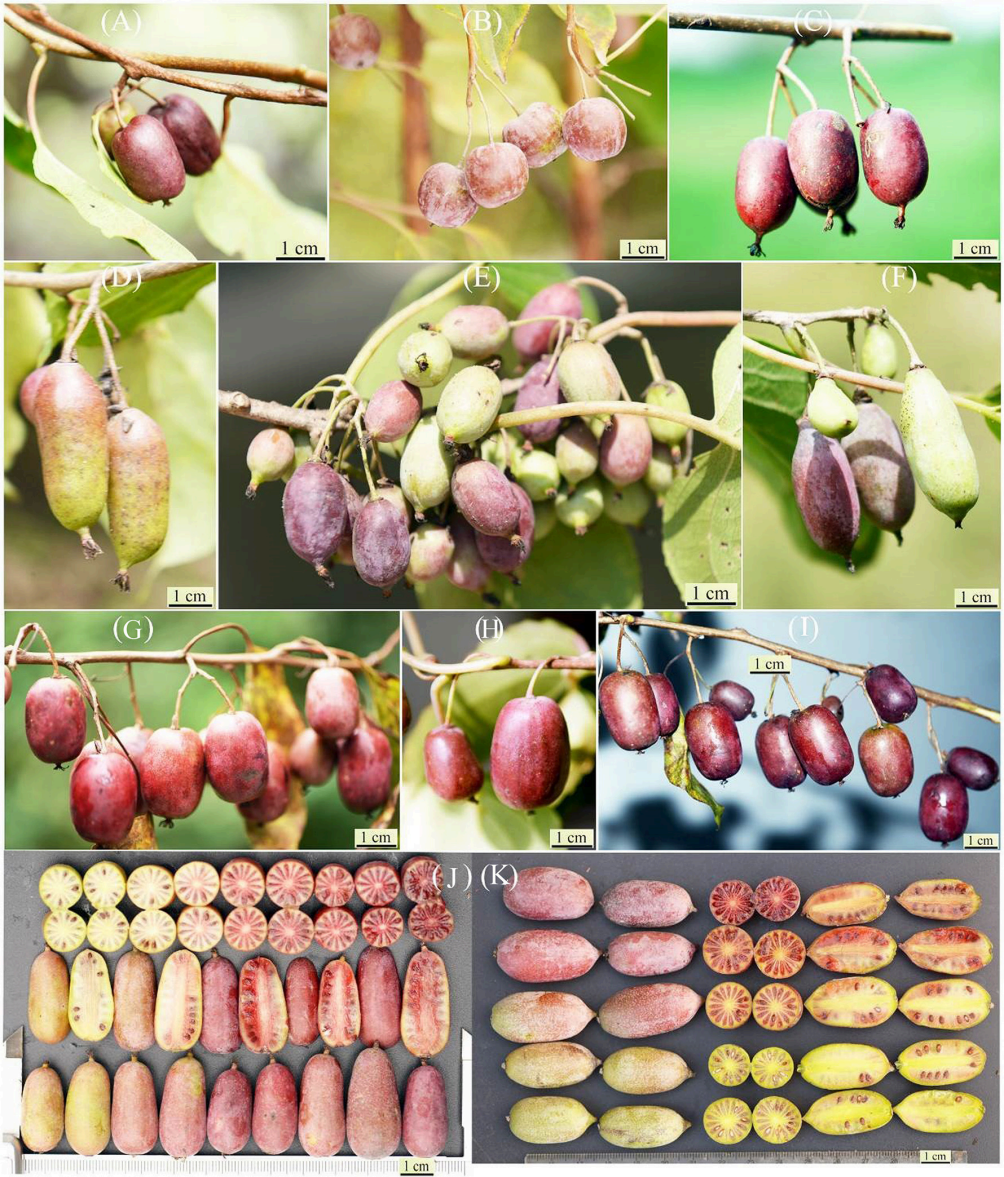
**Natural kiwiberries (*A. arguta* and related species) collected from the Qinling Mountain, China**. Fruits of **(A–C)** tetraploid *A. arguta* var. arguta, **(G–I)** tetraploid *A. melanandra*, **(D,E)** octaploid *A. arguta* var. *giraldii* **(F)** decaploid *A. arguta* var. giraldii, **(J,K)** elite germplasms selected from *A. arguta* var. *giraldii*.

The organic size is an important aspect of polyploids that has been associated with crop yield. A total of 8 quantitative characters including leaf length and width, petiole length, flower diameter, fruit weight, length, greater, and less diameter were qualified in 119 samples at least 3 times. The measurement was carried out between 2011 and 2015 based on the following procedures. The second and third leaves on strong stems were chosen and over 30 leaves were measured in each sample using Vernier calipers (Table 2; Table S2). The diameters of 30 flowers per sample were also determined by Vernier calipers. Thirty-five fruits were randomly selected for size and average weight determination (Figure 2).

**Table 2:** **Dimensional variation of leaves, flowers, and fruit among *Actinidia arguta* var. *arguta, A. arguta* var. *giraldii*, and *A. melanandra***.

**Taxon (ploidy levels)**	**Fruit**				**Flower**	**Leaf**		
	**Length (mm)**	**Greater diameter (mm)**	**Lesser diameter (mm)**	**Weight (g)**	**Diameter**	**Length (mm)**	**Width (mm)**	**Petiole length**
*A. arguta* var. *arguta* (4*x*)	27.44 ± 6.35a	18.68 ± 2.51b	17.09 ± 1.82b	4.85 ± 1.63b	19.30 ± 1.8a	97.17 ± 13.45b	56.22 ± 11.00b	44.74 ± 9.69b
*A. arguta* var. *giraldii* (8*x* and 10*x*)	29.49 ± 6.24a	13.64 ± 1.99a	13.26 ± 1.57a	2.92 ± 1.27a	20.00 ± 1.2a	110.07 ± 13.67c	68.85 ± 15.17c	52.91 ± 12.80c
*A. melanandra* (4*x*)	29.07 ± 4.00a	21.36 ± 3.10c	19.66 ± 2.831c	6.71 ± 1.52c	19.23 ± 1.6a	88.67 ± 14.35a	44.24 ± 9.65a	38.41 ± 11.21a
*P value*	0.230^ns^	0^***^	0^***^	0^***^	0.113^ns^	0^***^	0^***^	0^***^

Values are given as the mean ± s.d. Values in rows marked with different letters are significantly different at p ≤ 0.05. (

*** *P < 0.001; ns, not significant)*.

Qualitative characteristic analysis is an important feature for yield the performance of kiwiberries. Therefore, the content of soluble solids, ascorbic acid, total sugar, total acid, and total amino acids in all samples were measured according to the National Standard Methods of China. Total acid content, expressed as percentage of citric acid, was determined after titrating to pH 8.2 ± 0.1 using 0.1 M NaOH (SAC, 2008). Soluble solids content was estimated as the mean of digital refractometer (Atago; Japan) readings taken of juice expressed from the 10 mm end caps removed from opposite ends of the fruits. Total sugar was determined by a direct titrimetric method using Fehling's reagent (SB/T 10203–1994). Ascorbic acid (vitamin C) content was estimated by titration using the colored oxidation/reduction indicator 2, 6-dichlorophenolindophenol (SAC, 1994). Total and amino acids were determined by the ninhydrin colorimetric method (Chinese standard GB/T 5009, 2003) using an automatic amino acid analyser (model 8800; Hitachi Ltd., Japan). Upon determining that, *A. arguta* var. *giraldii* had obviously higher content of total amino acids than *A. arguta* var. *arguta* and *A. melanandra*, the content of 17 amino acids were further measured. In addition, the fruit of 35 samples (14 samples of *A. arguta* var. *arguta*, 9 samples of *A. arguta* var. *giraldii* and 12 samples of *A*. *melanandra*) showed brilliant red-fleshed color, and their total anthocyanins were extracted by methanol/formic acid and analyzed by reversed-phase high-performance liquid chromatography (HPLC) followed the method described by Comeskey et al. (2009).

### Statistics

Statistical calculations were performed using IBM® SPSS® Statistics 20 software (IBM SPSS Inc., Chicago, IL, USA). All data were assessed for normality and homogeneity of variance (Kolmogorov–Smirnov test) prior to further analysis to fulfill the requirements of statistical analysis of variance. Differences between the fruit, flower and leaf characters of *A. arguta* var. *arguta, A. arguta* var. *giraldii*, and *A. melanandra* were evaluated using one-way ANOVA at *p* ≤ 0.05 and 0.01. When ANOVA was significant, the means were discriminated using Duncan's test. Correlations between ecological and climate factors vs. cytotypes' distribution and morphological/fruit quality characters vs. ploidy levels were estimated using Pearson's correlation analysis.

## Results

### Cytotype variation and geographical distribution

Three DNA ploidy levels (4*x*, 8*x*, and 10*x*) were detected: *A*. *arguta* var. *giraldii* at 8*x* and 10*x*, and *A. arguta* var. *arguta* and *A. melanandra* at 4*x*. The most frequent cytotype was 4*x*, accounting for 76.47% of the samples studied (Table 1). Sympatric occurrence of *A. arguta* var. *arguta* (4*x*), *A. arguta* var. *giraldii* (8*x* and 10*x*) and *A. melanandra* (4*x*) was common in 16 sampled sites. In addition, all possible combination of cytotypes were found: 4*x* and 10*x* plants co-existed in the SZB, SMT and GHM populations; 4*x* and 8*x* plants in the HNS, HNT, and SHX populations; and 8*x* and 10*x* plants in the SHH and SBX populations.

**Figure 3:**
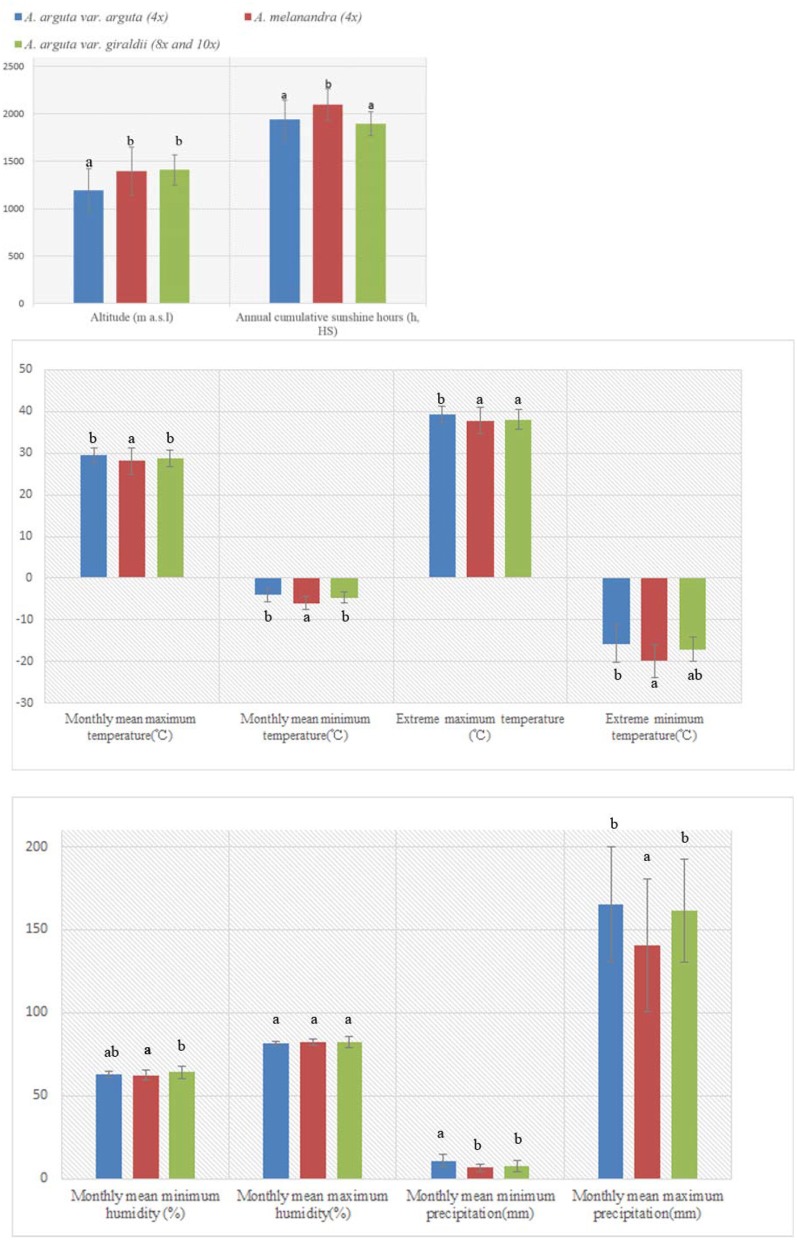
**The effect of climate and environment on *Actinidia arguta* var. *arguta*, *A. arguta* var. *giraldii* and *A. melanandra***.

The eco-geographical distribution of *A. arguta* var. *giraldii, A. arguta* var. *arguta*, and *A. melanandra* on multivariate vertical gradient, solar radiation, temperature, and precipitation are listed in Table S1 and depicted in Figure 1. Base on one-way ANOVA analysis, their eco-geographical distribution among three taxa had obvious difference of climate change, except for the maximum humidity (Figure 3). Of that, *A. arguta* var. *giraldii* (8*x* and 10*x*, 1,409 m asl) located at highest altitude, but it was not significantly different from *A. melanandra* (1,395 m). Furthermore, the individuals with higher ploidy levels (8*x* and 10*x*) did not show better adaptability for extreme temperature, higher solar radiation, lowest precipitation, and humidity. Similarity, Pearson correlation analysis demonstrated there was no significant relationship (*P* < 0.01) between ploidy levels and climate conditions (Table S3).

### The quantitative traits of kiwiberries' fruits, leaves, and flowers

Dimensional variation of leaves, flowers, and fruits among *Actinidia arguta* var. *arguta, A. arguta* var. *giraldii*, and *A. melanandra* are listed in Table 2. Octaploid and decaploid *A. arguta* var. *giraldii* had larger leaves than tetraploid *A. arguta* var. *arguta* and *A. melanandra*. However, fruit sizes of tetraploid *A. melanandra* were larger than *A. arguta* var. *arguta* which were, in turn, larger than those of octaploid and decaploid *A. arguta* var. *giraldii*. There was no significant difference between the flower sizes (*P* = 0.11 > 0.05) and fruit length (*P* = 0.23 > 0.05) among the three taxa. Pearson's correlation analysis confirmed that, ploidy level of three taxa had a negative relationship with fruit size (−0.67 < *r* < −0.53; *P* = 0 < 0.01), but positively correlated with leaf size (0.36 < *r* < 0.48; *P* = 0 < 0.01) (Table S4).

Analysis of the traits related to flavor and nutrition are shown in Table 3. Obvious variations were detected among the three taxa. The total sugar content varied from 6.38 to 8.67 g/100 g F.W., while the total acid content varied from 0.89 to 1.17 g/100 g F.W. The soluble solids content ranged from 12.85 to 13.88, which did not show significant difference (*P* = 0.092 > 0.05). The highest contents of soluble solids, total sugar, and total acid content were observed in fruit of *A. arguta* var. *arguta*, while the lowest were observed in *A. arguta* var. *giraldii*.

**Table 3:** **Concentrations of total sugars, soluble solids, total anthocyanin, total acids, ascorbic acid, and amino acids in fruit of *Actinidia arguta* var. *arguta, A. arguta* var. *giraldii*, and *A. melanandra* collected from the Qinling Mountain, China (g/100 g fresh weight)**.

**Species**	**Total sugar**	**Soluble solids content (%)**	**Total anthocyanin**	**Total acid**	**Ascorbic acid**	**Total amino acid**
*A. arguta* var. *arguta*	8.66336 ± 1.5416b	13.88 ± 2.15a	1.61 ± 1.11	1.1683 ± 0.2754a	0.0518 ± 0.0186a	0.9298 ± 0.1958a
*A. arguta* var. *giraldii*	6.3786 ± 1.9987a	12.85 ± 1.91a	6.06 ± 2.18	0.8939 ± 0.1618a	0.0889 ± 0.0366b	1.6338 ± 0.3570b
*A. melanandra*	8.5785 ± 0.9452b	13.68 ± 2.01a	6.82 ± 2.67	1.0759 ± 0.2694b	0.07871 ± 0.016b	0.9522 ± 0.0959a
*P*-value	0^***^	0.092^ns^	0.02^**^	0^***^	0^***^	0^***^

*Values are given as the mean ± s.d. Values in rows marked with different letters are significantly different at p ≤ 0.05*.

**, *** *, at P < 0.01, 0.001, respectively; ns, not significant*.

**Figure 4:**
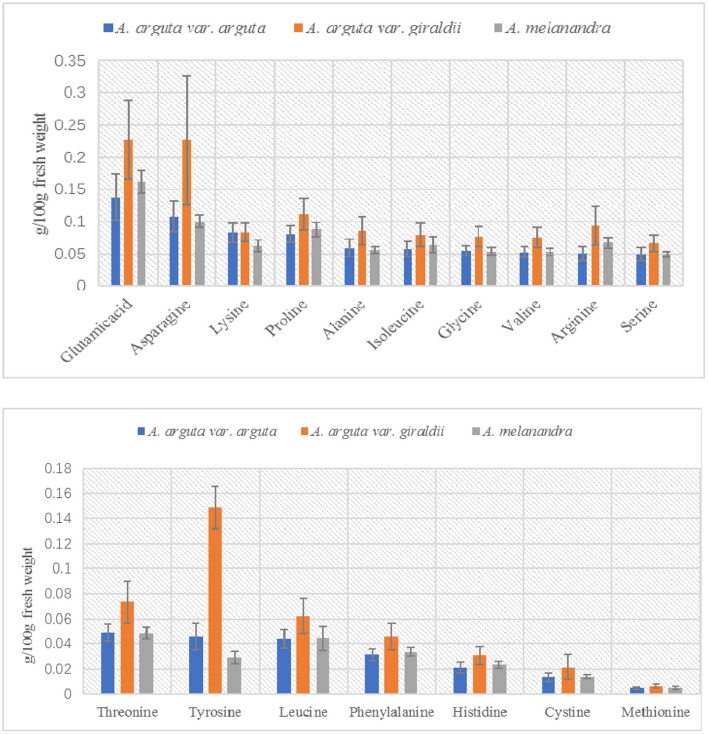
**The differentiation of 17 amino acids content among *Actinidia arguta* var. *arguta*, *A. arguta* var. *giraldii* and *A. melanandra***.

It is obvious that fruit of *A. arguta* var. *giraldii* contained more ascorbic acid and total amino acids than fruit from *A. arguta* var. *arguta* and *A. melanandra*. Ascorbic acid of three taxa varied significantly from 0.05 to 0.09 g/100 g F.W. and the total amino acids varied from 0.93 to 1.63 g/100 g F.W. In particular, the contents of 17 amino acids in octaploid and decaploid *A. arguta* var. *giraldii* were generally higher than that in other two tetraploid taxa in present study (Table 3; Figure 4). Moreover, high standard deviations (e.g., total sugar in *A. arguta* var. *giraldii*: 6. 3786 ± 1.998; Table 3) within taxa indicate high intra-variability in natural kiwiberries.

### Morphological and phenological variation

Fruit morphology of three taxa examined was highly variable both in the shape and color. *A. arguta* var. *arguta* predominantly had green fruit, whereas the fruits of *A. melanandra* and *A. arguta* var. *giraldii* were red or purple in color that differed in intensity (Figure 2). The average anthocyanins contents in *A. arguta* var. *giraldii* (6.11 mg/100 g F. W.) and *A. melanandra* (6.82 mg/100 g F. W.) are higher than *A. arguta* var. *arguta* (1.61 mg/100 g F. W.) in studied samples. Fruit shape of *A. arguta* var. *arguta* and *A. melanandra* (including ovoid, round, globose, oblong, and ellipsoidal) was more variable than *A. arguta* var. *giraldii*, whose fruit was similar in length but much leaner (Table 2).

Leaf shape and texture were similar in all three taxa. There was some micro-variation in the characteristics of the lower surface of the leaves (Figure S1). First, the lower surface of *A. melanandra* leaves were glaucous (Figure S1B), while this covering was almost completely absent from the leaves of both *A. arguta* var. *arguta* (Figure S1A) and *A. arguta* var. *giraldii* (Figures S1C,D). Secondly, the mid-vein on the lower surface of the leaves of *A. arguta* var. *giraldii* had a curly tomentum (Figures S1C,D), which was absent or very sparse on leaves of *A. arguta* var. *arguta* and *A. melanandra*.

There was no significant difference between the flower morphology of the three taxa (Figure S2). The flowers typically had white to light green petals, white filaments, and dark brown or black anthers. However, there were significant differences in phenology (Figure S3). *A. arguta* var. *arguta* (4*x*) and *A. melanandra* (4*x*) flowered in late April, partially overlapping from 2014 to 2016, whereas *A. arguta* var. *giraldii* (8*x* and 10*x*) flowered much later in mid-May. The ploidy races (8*x* and 10*x*) of *A. arguta* var. *giraldii* did not differ significantly in flowering time.

## Discussion

### The ploidy variations, distribution patterns and co-existent mechanisms

High-throughput ploidy analyses based on flow cytometry have revolutionized the study of ploidy variations and cyto-geography of *Actinidia* species (Ollitrault-Sammarcelli et al., 1994; Yan et al., 1997; Li et al., 2010), the chromosomes of which are particularly small and numerous (e.g., decaploid, 2*n* = 10*x* = 290). Octoploids and decaploids were initially discovered in natural *A. arguta* var. *giraldii* var. *giraldii*, which scattered in Qinling Mountains. Tetraploid *A. arguta* var. *giraldii* var. *arguta* and *A. melanandra* are the predominant ploidy races (76.47%), consistent with previous studies in Japan (Kataoka et al., 2010) and China (Li et al., 2013). Diploid kiwiberry (*A*. *hypoleuca*) was documented in Japan (Watanabe et al., 1990; Kataoka et al., 2010) that normally localized in relatively warm Pacific regions. Surprisingly, diploid species, as ancestors of polyploid races, were absented from the distributional areas of *A. arguta* var. *giraldii* and *A. melanandra*. The competitive exclusion could lead to their elimination from sympatric areas by polyploid progenies, who frequently grow larger and faster, with higher yields and better resistance to disease (Fowler and Levin, 1984; Te Beest et al., 2012; Renny-Byfield and Wendel, 2014). To sum up, we cautiously concluded that natural kiwiberries (*A. arguta* var. *giraldii* and related species) has the ploidy levels as following: *A*. *hypoleuca*-2*x, A. melanandra*- 4*x, A. arguta* var. *giraldii* var. *arguta*-4*x*, 6*x*, 7*x*, and 8*x, A. arguta* var. *giraldii* var. *giraldii* −8*x* and 10*x*.

The distributional pattern in sympatric areas of *A. arguta* var. *giraldii* var. *arguta, A. arguta* var. *giraldii* var. *giraldii*, and *A. melanandra* is characterized by a high frequency of mixed-ploidy populations (56.25%). The cytotype mixture was considered to be an evolutionarily unstable pattern, likely reflecting *in situ* formation or frequent cytotype immigration, consistent with the minority cytotype exclusion model (Levin, 1975), resulting in the elimination of the minority cytotypes (Baack, 2005; Zozomová-Lihová et al., 2015). Recent studies, however, have shown that mixed-cytotype populations are frequent and that balancing selection is commonly observed in these populations (Burton and Husband, 1999; Keeler, 2004; Kao, 2007; Castro et al., 2012; Duchoslav et al., 2016). Theoretical studies suggest that the long-term sympatric growth of cytotypes can only be maintained when different ploidy races have strong post- or prezygotic isolation mechanisms (Levin, 1975; Rodríguez, 1996). In the present study, no obvious niche differentiation was observed within the population to balance the spatial segregation of polyploidy species. However, over the three years studied, tetraploids consistently flowered much earlier than octaploids and decaploids (Figure S3). Divergence in flowering time is a by-product of natural selection, potentially resulting in reproductive isolation to maintain the co-existence of cytotypes (Van Dijk and Bijlsma, 1994; Petit et al., 1997); in the present study, the prezygotic isolation mechanism also played an important role in the co-existence of cytotypes in the 4*x* and 8*x* (HNA and HNT) and 4*x* and 10*x* populations (SZB and GHM).

### The plus, the better? higher ploidy level vs. ecological adaption and agnomical traits

Polyploids are deem to be more resistant to extreme condition (Stebbins, 1985; Brochmann et al., 2004), such as higher altitude, cold, heat, or drought stress, which lead them more easier to invasion of new habitats (Te Beest et al., 2012). For example, a systematic investigation of the allopolyploid, autopolyploid, and diploid hybrid species along an elevation gradient from sea level to 4,500 m within British Columbia, Canada, provided evidence that polyploids were disproportionately present at high elevations (Vamosi and McEwen, 2012). Previous studies within *Actinidia* genus have shown that hexaploid *A*. *chinensis* var. *deliciosa* plants in China grow at higher altitudes than both tetraploid and diploid *A*. *chinensis* var. *chinensis* plants (Li et al., 2010) and hexaploid *A. arguta* var. *giraldii* var. *arguta* plants are geographically localized in the colder regions of Japan, whereas diploid plants of the closely related *A. hypoleuca* are located in warmer regions (Kataoka et al., 2010). In this study, *A. arguta* var. *giraldii* var. *giraldii*, with higher ploidy levels (8*x* and 10*x*), were scattered in higher altitude than *A. arguta* var. *giraldii* var. *argute*, but the difference is no significant with tetraploid *A. melanandra*. In particular, the tetraploid *A. melanandra* could survived in more challenging climate, extreme temperature and few precipitation for instance (Figure 2). With diversified species, the tetraploid accounts for the highest proportion of ploidy races in natural kiwiberries, and successfully colonize different environments (Kataoka et al., 2010; Li et al., 2013). The adaptability of kiwiberries to harsh environments and climates, therefore, could not consistently enhance in response to the elevated ploidy levels.

The association between ploidy levels and morphological or quality characteristics is certainly complex in *Actinidia* genus. Studies on ploidy manipulation further confirmed that the fruit of colchicine-induced autotetraploids of *A. chinensis* were 50 to 60% larger than those of their diploid progenitors (Wu et al., 2012, 2013). A previous study on *A. arguta* var. *giraldii* and related species confirmed larger leaf and fruit sizes in the tetraploid and hexaploid fruit of *A. arguta* var. *giraldii* in Japan (Kataoka et al., 2010). In the present study, the leaf size was obviously larger in these individuals and positively correlated with higher ploidy levels (8*x* and 10*x*). Particularly, the nutritional ingredients of *A. arguta* var. *giraldii* var. *giraldii* (8*x* and 10*x*), including the amount of ascorbic acid and amino acids, were much higher than tetraploid *A. arguta* var. *giraldii* var. *arguta* and *A. melanandra*, implying that *A. arguta* var. *giraldii* var. *giraldii* could serve as a useful germplasm to attain rapid genetic improvement with respect to improved nutritional ingredients. However, the disadvantage of high ploidy races should be highlighted to scientists and breeders, as the fruit shape significantly varied after polyploidization of diploid *A*. *chinensis* (Wu et al., 2012). In addition, reduced flesh firmness and dry matter and less intense golden flesh color were observed in autotetraploid plants compared with parental diploid plants (Wu et al., 2013). In the present study, the fruit size of *A. arguta* var. *giraldii* var. *giraldii* decreased with the increasing ploidy level (8*x* and 10*x*), and some commercially important characteristics associated with fruit quality (e.g., total sugar and total acid content) was also poor on 8*x* and 10*x* plants. Therefore, the morphological and quality characteristics were not necessarily positively and linearly correlated with continuously increasing ploidy levels (Table S4). Thus, higher ploidy is not inevitably better in the *Actinidia* genus.

### Conclusion and breeding implication

Studies of the natural resources of *A. arguta* var. *giraldii* and related species have enhanced our current understanding of ploidy variation, distributional pattern, and co-existence mechanisms of cytotypes. The establishment of a genetic diversity center of *A. arguta* var. *giraldii* and related species near Qinling Mountain, where species with abundant ploidy variations and diversified phenotypes presenting colorful fruit fresh, variable fruit shape, different fruit size, and nutritional compositions are detected (Figure 2), would be advantageous. The relationship between ploidy levels and agronomic traits, such as the polyploid advantage on a higher content of ascorbic acid and amino acids, and the disadvantages regarding ecological adaptation, fruit size, and fruit flavor, will improve our knowledge of multi-polyploidization in plants.

Substantially, the present study of new genotypes, including higher nutrient content, edible skins, colorful fruit flesh (red, purple) and new flavors, is the first step in germplasm exploration, and we expect to extend the utility of such genetic material to ultimately improve traditional kiwifruit quality, which is characterized by brown hairy skin, green flesh and acid flavor. To achieve task, symmetrical large-scale surveys, and detailed evaluations (solid soluble content, dry matter, aroma, yield, etc.) of natural germplasms planted in kiwifruit orchards are needed. Similarly, whole genome re-sequencing of *A. arguta var. giraldii* var. *arguta, A. arguta var. giraldii* var. *giraldii*, and *A. melanandra* should be conducted, although there may be particular challenges for analyzing improvement traits, reflecting the high genomic heterozygosity and inadequacies of the analytic techniques used to examine polyploidy. We anticipate that these methodological challenges will be overcome by advances in genome sequencing technologies (Faino and Thomma, 2014). Finally, other powerful approaches (e.g., CRISPR-Cas system) (Kanchiswamy et al., 2015) for the examination of kiwifruit will improve association studies examining the genotype vs. phenotype, an essential prerequisite to targeted breeding efforts.

## Author contributions

DL and YZ conceived and planned the study. YZ and CZ collected the materials and measured the traits of fruit, leaf and flower. XS provided materials and data of A. arguta var. arguta of North China. DL, YL, and QZ tested the ploidy levels. DL wrote the manuscript.

## Funding

This research is funded by the National Natural Science Foundation of China (Project no. 31572092) and the Agricultural Public Relations Project of Shaanxi Technology Committee (Project no. 2014K01-07-02).

### Conflict of interest statement

The authors declare that the research was conducted in the absence of any commercial or financial relationships that could be construed as a potential conflict of interest.

## References

[B1] Adams K. L. Wendel J. F. (2005). Polyploidy and genome evolution in plants. Curr. Opin. Plant Biol. 8, 135–141. 10.1016/j.pbi.2005.01.001 15752992

[B2] Asakura I. Hoshino Y. (2016). Distribution, ploidy levels, and fruit characteristics of three *Actinidia* species native to Hokkaido, Japan. Horticult. J. 85, 105–114. 10.2503/hortj.MI-082

[B3] Baack E. J. (2005). Ecological factors influencing tetraploid establishment in snow buttercups (Ranunculus adoneus, Ranunculaceae): minority cytotype exclusion and barriers to triploid formation. Am. J. Bot. 92, 1827–1835. 10.3732/ajb.92.11.1827 21646100

[B4] Bieniek A. (2012). Yield, morphology and biological value of fruits of *Actinidia arguta* and Actinidia purpurea and some of their hybrid cultivars grown in north-eastern Poland. Acta. Sci. Pol. Hortorum. Cultus 11, 117–130.

[B5] Boyd L. McNeilage M. Macrae E. Ferguson A. Beatson R. Martin P. (2002). Development and commercialization of 'baby kiwi'(*Actinidia arguta* Planch.), in V International Symposium on Kiwifruit (Wuhan).

[B6] Brochmann C. Brysting A. Alsos I. Borgen L. Grundt H. Scheen A. C. (2004). Polyploidy in arctic plants. Biol. J. Linn. Soc. 82, 521–536. 10.1111/j.1095-8312.2004.00337.x

[B7] Burton T. L. Husband B. C. (1999). Population cytotype structure in the polyploid Galax urceolata (Diapensiaceae). Heredity 82, 381–390. 10.1038/sj.hdy.6884910 10383656

[B8] Castro S. Loureiro J. Procházka T. Münzbergová Z. (2012). Cytotype distribution at a diploid–hexaploid contact zone in Aster amellus (Asteraceae). Ann. Bot. 110, 1047–1055. 10.1093/aob/mcs177 22887024 PMC3448430

[B9] Cheng S. H. Zhuang J. Y. Fan Y. Y. Du J. H. Cao L. Y. (2007). Progress in research and development on hybrid rice: a super-domesticate in China. Ann. Bot. 100, 959–966. 10.1093/aob/mcm121 17704538 PMC2759200

[B10] Chinese standard GB/T 5009 (2003). Chinese Standard GB/T 5009.124-2003, 2004. Inspection of Grain and Oilseeds: Method for Determination of Amino Acids in Foods. Beijing: Standards Press of China.

[B11] Comai L. (2005). The advantages and disadvantages of being polyploid. Nat. Rev. Genet. 6, 836–846. 10.1038/nrg1711 16304599

[B12] Comeskey D. J. Montefiori M. Edwards P. J. B. McGhie T. K. (2009). Isolation and structural identification of the anthocyanin components of red kiwifruit. Agric. Food Chem. 57, 2035–2039. 10.1021/jf803287d 19203266

[B13] Crow J. F. (1998). 90 years ago: the beginning of hybrid maize. Genetics 148, 923–928. 9539413 10.1093/genetics/148.3.923 PMC1460037

[B14] Datson P. Ferguson A. (2011). Actinidia, in Wild Crop Relatives: Genomic and Breeding Resources, ed Kole C. (Berlin; Heidelberg: Springer), 1–20.

[B15] Datson P. Nardozza S. Manako K. Herrick J. Martinez-Sanchez M. Curtis C. (2013). Monitoring the Actinidia germplasm for resistance to *Pseudomonas syringae* pv. *Actinidiae*, in I International Symposium on Bacterial Canker of Kiwifruit (Mt Maunganui).

[B16] Dhawan O. Lavania U. (1996). Enhancing the productivity of secondary metabolites via induced polyploidy: a review. Euphytica 87, 81–89. 10.1007/BF00021879

[B17] Dubcovsky J. Dvorak J. (2007). Genome plasticity a key factor in the success of polyploid wheat under domestication. Science 316, 1862–1866. 10.1126/science.1143986 17600208 PMC4737438

[B18] Duchoslav M. Fialová M. Jandová M. (2016). The ecological performance of tetra-, penta-and hexaploid geophyte *Allium oleraceum* in reciprocal transplant experiment may explain the occurrence of multiple-cytotype populations. J. Plant Ecol. rtw039 10.1093/jpe/rtw039

[B19] Duvick D. N. (2001). Biotechnology in the 1930s: the development of hybrid maize. Nat. Rev. Genet. 2, 69–74. 10.1038/35047587 11253074

[B20] Faino L. Thomma B. P. (2014). Get your high-quality low-cost genome sequence. Trends Plant Sci. 19, 288–291. 10.1016/j.tplants.2014.02.003 24636622

[B21] Ferguson A. (2007). The need for characterisation and evaluation of germplasm: kiwifruit as an example. Euphytica 154, 371–382. 10.1007/s10681-006-9188-2

[B22] Ferguson A. Huang H. (2007). Genetic resources of kiwifruit: domestication and breeding. Hortic. Rev. 33, 1–121. 10.1002/9780470168011.ch1

[B23] Ferguson A. Seal A. (2008). Kiwifruit, in Temperate Fruit Crop Breeding, ed Hancock J. F. (Springer), 235–264.

[B24] Fowler N. L. Levin D. A. (1984). Ecological constraints on the establishment of a novel polyploid in competition with its diploid progenitor. Am. Nat. 124, 703–711. 10.1086/284307

[B25] Hijmans R. J. Gavrilenko T. Stephenson S. Bamberg J. Salas A. Spooner D. M. (2007). Geographical and environmental range expansion through polyploidy in wild potatoes (Solanum section Petota). Glob. Ecol. Biogeogr. 16, 485–495. 10.1111/j.1466-8238.2007.00308.x

[B26] Kagan-Zur V. Yaron-Miron D. Mizrahi Y. (1991). A study of triploid tomato fruit attributes. J. Am. Chem. Soc. 116, 228–231.

[B27] Kanchiswamy C. N. Sargent D. J. Velasco R. Maffei M. E. Malnoy M. (2015). Looking forward to genetically edited fruit crops. Trends Biotechnol. 33, 62–64. 10.1016/j.tibtech.2014.07.003 25129425

[B28] Kao R. H. (2007). Asexuality and the coexistence of cytotypes. New Phytol. 175, 764–772. 10.1111/j.1469-8137.2007.02145.x 17688591

[B29] Kataoka I. Mizugami T. Kim J. G. Beppu K. Fukuda T. Sugahara S. (2010). Ploidy variation of hardy kiwifruit (*Actinidia arguta*) resources and geographic distribution in Japan. Sci. Hortic. 124, 409–414. 10.1016/j.scienta.2010.01.016

[B30] Keeler K. H. (2004). Impact of intraspecific polyploidy in Andropogon gerardii (Poaceae) populations. Am. Midl. Nat. 152, 63–74. 10.1674/0003-0031(2004)152[0063:IOIPIA]2.0.CO;2

[B31] King K. Seppälä O. Neiman M. (2012). Is more better? Polyploidy and parasite resistance. Biol. Lett. 8, 598–600. 10.1098/rsbl.2011.1152 22258448 PMC3391438

[B32] Lavania U. C. (2013). Polyploidy, body size, and opportunities for genetic enhancement and fixation of heterozygosity in plants. Nucleus 56, 1–6. 10.1007/s13237-013-0075-7

[B33] Lavania U. C. Srivastava S. Lavania S. Basu S. Misra N. K. Mukai Y. (2012). Autopolyploidy differentially influences body size in plants, but facilitates enhanced accumulation of secondary metabolites, causing increased cytosine methylation. Plant J. 71, 539–549. 10.1111/j.1365-313X.2012.05006.x 22449082

[B34] Leitch A. Leitch I. (2008). Genomic plasticity and the diversity of polyploid plants. Science 320, 481–483. 10.1126/science.1153585 18436776

[B35] Levin D. A. (1975). Minority cytotype exclusion in local plant populations. Taxon 24, 35–43. 10.2307/1218997

[B36] Levin D. A. (2002). The Role of Chromosomal Change in Plant Evolution. New York, NY: Oxford University Press.

[B37] Li D. Liu Y. Zhong C. Huang H. (2010). Morphological and cytotype variation of wild kiwifruit (*Actinidia chinensis* complex) along an altitudinal and longitudinal gradient in central-west China. Biol. J. Linn. Soc. 164, 72–83. 10.1111/j.1095-8339.2010.01073.x

[B38] Li J. Li X. Soejarto D. (2007). Actinidiaceae. Flora China 12, 334–360.

[B39] Li X. Li J. Soejarto D. D. (2007). New synonyms in Actinidiaceae from China. Acta Phylotaxon Sin. 45, 633–660. 10.1360/aps06061

[B40] Li Z. Z. Man Y. P. Lan X. Y. Wang Y. C. (2013). Ploidy and phenotype variation of a natural *Actnidia arguta* population in the east of Daba Mountain located in a region of Shaanxi. Sci. Hortic. 161, 259–265. 10.1016/j.scienta.2013.07.008

[B41] Luo Y. Yen X. Zhang G. Liang G. (1992). Agronomic traits and chromosome behavior of autotetraploid sorghums. Plant Breed. 109, 46–53. 10.1111/j.1439-0523.1992.tb00149.x

[B42] Matich A. J. Young H. Allen J. M. Wang M. Y. Fielder S. McNeilage M. A.. (2003). *Actinidia arguta*: volatile compounds in fruit and flowers. Phytochemistry 63, 285–301. 10.1016/S0031-9422(03)00142-0 12737978

[B43] Nishiyama I. Fukuda T. Oota T. (2005). Genotypic differences in chlorophyll, lutein, and β-carotene contents in the fruits of Actinidia species. J. Agr. Food Chem. 53, 6403–6407. 10.1021/jf050785y 16076125

[B44] Nishiyama I. Fukuda T. Shimohashi A. Oota T. (2008). Sugar and organic acid composition in the fruit juice of different Actinidia varieties. Food Sci. Technol. Res. 14, 67–73. 10.3136/fstr.14.67

[B45] Ollitrault-Sammarcelli F. Legave J. Michaux-Ferriere N. Hirsch A. M. (1994). Use of flow cytometry for rapid determination of ploidy level in the genus Actinidia. Sci. Hortic. 57, 303–313. 10.1016/S0304-4238(94)90113-9

[B46] Otto S. P. Whitton J. (2000). Polyploid incidence and evolution. Annu. Rev. Genet. 34, 401–437. 10.1146/annurev.genet.34.1.401 11092833

[B47] Petit C. Lesbros P. Ge X. Thompson J. D. (1997). Variation in flowering phenology and selfing rate across a contact zone between diploid and tetraploid *Arrhenatherum elatius* (Poaceae). Heredity 79, 31–40. 10.1038/hdy.1997.120

[B48] Renny-Byfield S. Wendel J. F. (2014). Doubling down on genomes: polyploidy and crop plants. Am. J. Bot. 101, 1711–1725. 10.3732/ajb.1400119 25090999

[B49] Rodríguez D. J. (1996). Model for the establishment of polyploidy in plants: viable but infertile hybrids, iteroparity, and demographic stochasticity. J. Theor. Biol. 3, 189–196. 10.1006/jtbi.1996.0095

[B50] SAC (1994). Determination of Vitamin C in Vegetables and Fruits (2. 6- dichloro-indophenol titration. Beijing: Standards Press of China.

[B51] SAC (2008). Determination of Total Acid in Foods. Vol. GB/T 12456-2008, Standardization Administration of the People's Republic of China. Beijing: Standards Press of China.

[B52] Soltis D. E. Albert V. A. Leebens-Mack J. Bell C. D. Paterson A. H. Zheng C.. (2009). Polyploidy and angiosperm diversification. Am. J. Bot. 96, 336–348. 10.3732/ajb.0800079 21628192

[B53] Soltis D. E. Buggs R. Doyle J. J. Soltis P. S. (2010). What we still don't know about polyploidy. Taxon 59, 1387–1403. 10.2307/20774036

[B54] Sonnleitner M. Hülber K. Flatscher R. García P. E. Winkler M. Suda J.. (2016). Ecological differentiation of diploid and polyploid cytotypes of Senecio carniolicus sensu lato (Asteraceae) is stronger in areas of sympatry. Ann. Bot. 117, 269–276. 10.1093/aob/mcv176 26658487 PMC4724049

[B55] Stebbins G. L. (1985). Polyploidy, hybridization, and the invasion of new habitats. Ann. Mo. Bot. Gard. 72, 824–832. 10.2307/2399224

[B56] Suda J. Weiss-Schneeweiss H. Tribsch A. Schneeweiss G. M. Trávníček P. Schönswetter P. (2007). Complex distribution patterns of di-, tetra-, and hexaploid cytotypes in the European high mountain plant Senecio carniolicus (Asteraceae). Am. J. Bot. 94, 1391–1401. 10.3732/ajb.94.8.1391 21636507

[B57] Te Beest M. Le Roux J. J. Richardson D. M. Brysting A. K. Suda J. Kubešová M.. (2012). The more the better? The role of polyploidy in facilitating plant invasions. Ann. Bot. 109, 19–45. 10.1093/aob/mcr277 22040744 PMC3241594

[B58] Udall J. A. Wendel J. F. (2006). Polyploidy and crop improvement. Crop Sci. 46, 3–14. 10.2135/cropsci2006.07.0489tpg

[B59] Uddin M. Ellison F. O'brien L. Latter B. (1992). Heterosis in F1 hybrids derived from crosses of adapted Australian wheats. Crop Pasture Sci. 43, 907–919. 10.1071/AR9920907

[B60] Vamosi J. C. McEwen J. R. (2012). Origin, elevation, and evolutionary success of hybrids and polyploids in British Columbia, Canada. Botany 91, 182–188. 10.1139/cjb-2012-0177

[B61] Van Dijk P. Bijlsma R. (1994). Simulations of flowering time displacement between two cytotypes that form inviable hybrids. Heredity 72, 522–535. 10.1038/hdy.1994.70

[B62] Wagner W. (1970). Biosystematics and evolutionary noise. Taxon 19, 146–151.

[B63] Warner D. A. Ku M. S. Edwards G. E. (1987). Photosynthesis, leaf anatomy, and cellular constituents in the polyploid C4 grass *Panicum virgatum*. Plant Physiol. 84, 461–466. 10.1104/pp.84.2.461 16665462 PMC1056603

[B64] Watanabe K. Takahashi B. Shirato K. (1990). Chromosome numbers in kiwifruit (*Actinidia deliciosa*) and related species. J. Jpn. Soc. Food Sci. 58, 835–840. 10.2503/jjshs.58.835

[B65] Wendel J. F. Cronn R. C. (2003). Polyploidy and the evolutionary history of cotton. Adv Agron. 78, 139–186. 10.1016/S0065-2113(02)78004-8

[B66] Wu J. H. Ferguson A. R. Murray B. G. Duffy A. M. Jia Y. Cheng C. (2013). Fruit quality in induced polyploids of *Actinidia chinensis*. HortScience 48, 701–707.

[B67] Wu J. H. Ferguson A. R. Murray B. G. Jia Y. Datson P. M. Zhang J. (2012). Induced polyploidy dramatically increases the size and alters the shape of fruit in *Actinidia chinensis*. Ann. Bot. 109, 169–179. 10.1093/aob/mcr256 21980192 PMC3241580

[B68] Yan G. Yao J. Ferguson A. McNeilage M. Seal A. Murray B. (1997). New reports of chromosome numbers in Actinidia (Actinidiaceae). New Zeal. J. Bot. 35, 181–186. 10.1080/0028825X.1997.10414154

[B69] Zozomová-Lihová J. Malánová-Krásná I. Vít P. Urfus T. Senko D. Svitok M.. (2015). Cytotype distribution patterns, ecological differentiation, and genetic structure in a diploid–tetraploid contact zone of Cardamine amara. Am. J. Bot. 102, 1380–1395. 10.3732/ajb.1500052 26290560

