# Supplementary material for: Agronomic Trait Variations and Ploidy Differentiation of Kiwiberries in Northwest China: Implication for Breeding
Source: Front Plant Sci. 2017 May 11;8:711. doi: 10.3389/fpls.2017.00711 (PMC5426280; doi:10.3389/fpls.2017.00711)
Supplement: Supplementary file 1 [file DataSheet1.pdf]

Supplement Table 1 The climate data of 16 populations of *A. arguta* var. *arguta*, *A. arguta* var. *giraldii* and *A. melanandra*

Species	Ploidy levels	Population code	Altitude (m a.s.l)	Annual cumulative sunshine hours (h, HS)	Monthly mean maximum temperature (℃)	Monthly mean minimum temperature (℃)	Monthly extreme maximum temperature (℃)	Monthly extreme minimum temperature (℃)	Monthly mean minimum humidity (% rh)	Monthly mean maximum humidity (% rh)	Monthly mean minimum precipitation (mm)	Monthly mean maximum precipitation (mm)
A. melanandra	Tetraploid	SZB	1424.00	1726.5	23.06	-7.74	33.76	-19.34	60.00	83.00	6.40	196.30
A. melanandra	Tetraploid	SZB	1516.33	1726.5	22.51	-8.29	33.21	-19.89	60.00	83.00	6.40	196.30
A. melanandra	Tetraploid	SZB	1608.67	1726.5	21.95	-8.85	32.65	-20.45	60.00	83.00	6.40	196.30
A. melanandra	Tetraploid	SZB	1701.00	1726.5	21.40	-9.40	32.10	-21.00	60.00	83.00	6.40	196.30
A. arguta var. *giraldii*	Decaploid	SZB	1385.00	1726.5	23.30	-7.50	34.00	-19.10	60.00	83.00	6.40	196.30
A. melanandra	Tetraploid	SSH	1371.00	2312.6	28.60	-6.40	38.60	-22.60	61.00	81.00	8.50	127.30
A. melanandra	Tetraploid	SSH	1372.00	2312.6	28.59	-6.41	38.59	-22.61	61.00	81.00	8.50	127.30
A. melanandra	Tetraploid	SSH	1373.00	2312.6	28.59	-6.41	38.59	-22.61	61.00	81.00	8.50	127.30
A. melanandra	Tetraploid	SSH	1374.00	2312.6	28.58	-6.42	38.58	-22.62	61.00	81.00	8.50	127.30
A. melanandra	Tetraploid	SSH	1375.00	2312.6	28.58	-6.42	38.58	-22.62	61.00	81.00	8.50	127.30
A. melanandra	Tetraploid	SSH	1376.00	2312.6	28.57	-6.43	38.57	-22.63	61.00	81.00	8.50	127.30
A. melanandra	Tetraploid	SSH	1378.00	2312.6	28.56	-6.44	38.56	-22.64	61.00	81.00	8.50	127.30
A. arguta var. *arguta*	Tetraploid	SNT	601.00	1726.5	29.20	-3.10	37.40	-16.40	70.00	87.00	6.20	193.90
A. arguta var. *arguta*	Tetraploid	SNT	648.50	1726.5	27.72	-3.09	38.42	-14.69	60.00	83.00	6.40	196.30
A. arguta var. *arguta*	Tetraploid	SNT	696.00	1726.5	27.43	-3.37	38.13	-14.97	60.00	83.00	6.40	196.30
A. melanandra	Tetraploid	SMT	1260.00	2015.2	25.50	-7.90	34.00	-29.80	60.00	82.00	4.00	140.30
A. melanandra	Tetraploid	SMT	1300.00	2015.2	25.26	-8.14	33.76	-30.04	60.00	82.00	4.00	140.30
A. arguta var. *arguta*	Tetraploid	SMT	1260.00	2015.2	28.00	-2.80	38.70	-14.40	60.00	83.00	6.40	196.30
A. arguta var. *arguta*	Tetraploid	SMT	1270.00	2015.2	27.94	-2.86	38.64	-14.46	60.00	83.00	6.40	196.30
A. arguta var. *arguta*	Tetraploid	SMT	1280.00	2015.2	25.38	-8.02	33.88	-29.92	60.00	82.00	4.00	140.30
A. arguta var. *arguta*	Tetraploid	SMT	1290.00	2015.2	25.32	-8.08	33.82	-29.98	60.00	82.00	4.00	140.30
A. arguta var. *arguta*	Tetraploid	SMT	1300.00	2015.2	25.26	-8.14	33.76	-30.04	60.00	82.00	4.00	140.30
A. arguta var. *giraldii*	Decaploid	SMT	1285.00	2015.2	25.35	-8.05	33.85	-29.95	60.00	82.00	4.00	140.30
A. melanandra	Tetraploid	SLQ	1200.00	2312.6	28.51	-6.49	38.51	-22.69	61.00	81.00	8.50	127.30
A. arguta var. *arguta*	Tetraploid	SLQ	1185.00	2312.6	28.60	-6.40	38.60	-22.60	61.00	81.00	8.50	127.30
A. arguta var. *arguta*	Tetraploid	SLQ	1236.25	2312.6	28.29	-6.71	38.29	-22.91	61.00	81.00	8.50	127.30
A. arguta var. *arguta*	Tetraploid	SLQ	1287.50	2312.6	27.99	-7.02	37.99	-23.22	61.00	81.00	8.50	127.30
A. arguta var. *arguta*	Tetraploid	SLQ	1338.75	2312.6	27.68	-7.32	37.68	-23.52	61.00	81.00	8.50	127.30
A. arguta var. *arguta*	Tetraploid	SLQ	1390.00	2312.6	27.37	-7.63	37.37	-23.83	61.00	81.00	8.50	127.30
A. melanandra	Tetraploid	SLG	1406.00	2350.00	32.50	-4.40	42.80	-16.70	63.00	82.00	5.50	95.00
A. melanandra	Tetraploid	SLG	1406.80	2350.00	32.50	-4.40	42.80	-16.70	63.00	82.00	5.50	95.00
A. melanandra	Tetraploid	SLG	1407.60	2350.00	32.51	-4.41	42.79	-16.71	63.00	82.00	5.50	95.00

A. melanandra	Tetraploid	SLG	1408.40	2350.00	32.51	-4.41	42.79	-16.71	63.00	82.00	5.50	95.00
A. melanandra	Tetraploid	SLG	1409.20	2350.00	32.52	-4.42	42.78	-16.72	63.00	82.00	5.50	95.00
A. melanandra	Tetraploid	SLG	1410.00	2350.00	32.52	-4.42	42.78	-16.72	63.00	82.00	5.50	95.00
A. arguta var. *arguta*	Tetraploid	SLG	1406.00	2350.00	32.50	-4.40	42.80	-16.70	63.00	82.00	5.50	95.00
A. arguta var. *arguta*	Tetraploid	SLG	1406.57	2350.00	32.50	-4.40	42.80	-16.70	63.00	82.00	5.50	95.00
A. arguta var. *arguta*	Tetraploid	SLG	1407.14	2350.00	32.51	-4.41	42.79	-16.71	63.00	82.00	5.50	95.00
A. arguta var. *arguta*	Tetraploid	SLG	1407.71	2350.00	32.51	-4.41	42.79	-16.71	63.00	82.00	5.50	95.00
A. arguta var. *arguta*	Tetraploid	SLG	1408.28	2350.00	32.51	-4.41	42.79	-16.71	63.00	82.00	5.50	95.00
A. arguta var. *arguta*	Tetraploid	SLG	1408.85	2350.00	32.52	-4.42	42.78	-16.72	63.00	82.00	5.50	95.00
A. arguta var. *arguta*	Tetraploid	SLG	1409.42	2350.00	32.52	-4.42	42.78	-16.72	63.00	82.00	5.50	95.00
A. arguta var. *arguta*	Tetraploid	SLG	1410.00	2350.00	32.52	-4.42	42.78	-16.72	63.00	82.00	5.50	95.00
A. arguta var. *giraldii*	Octaploid	SHX	1550.00	1850.00	27.19	-5.11	35.39	-18.41	70.00	87.00	6.20	193.90
A. melanandra	Tetraploid	SHX	1500.00	1850.00	27.49	-4.81	35.69	-18.11	70.00	87.00	6.20	193.90
A. melanandra	Tetraploid	SHX	1550.00	1850.00	27.19	-5.11	35.39	-18.41	70.00	87.00	6.20	193.90
A. arguta var. arguta	Tetraploid	SHX	1500.00	1850.00	27.49	-4.81	35.69	-18.11	70.00	87.00	6.20	193.90
A. arguta var. arguta	Tetraploid	SHX	1672.00	1850.00	26.46	-5.84	34.66	-19.14	70.00	87.00	6.20	193.90
A. arguta var. *giraldii*	Octaploid	SHH	1215.00	1850.00	29.20	-3.10	37.40	-16.40	70.00	87.00	6.20	193.90
A. arguta var. *giraldii*	Octaploid	SHH	1283.00	1850.00	28.79	-3.51	36.99	-16.81	70.00	87.00	6.20	193.90
A. arguta var. *giraldii*	Octaploid	SHH	1351.00	1850.00	28.38	-3.92	36.58	-17.22	70.00	87.00	6.20	193.90
A. arguta var. *giraldii*	Octaploid	SHH	1419.00	1850.00	27.98	-4.32	36.18	-17.62	70.00	87.00	6.20	193.90
A. arguta var. *giraldii*	Decaploid	SHH	1317.00	1850.00	28.59	-3.71	36.79	-17.01	70.00	87.00	6.20	193.90
A. arguta var. *giraldii*	Octaploid	SCP	1545.00	1860.2	29.70	-4.70	39.00	-17.50	60.00	82.00	5.10	158.70
A. arguta var. *giraldii*	Octaploid	SCP	1566.00	1860.2	29.57	-4.83	38.87	-17.63	60.00	82.00	5.10	158.70
A. arguta var. *giraldii*	Octaploid	SCP	1587.00	1860.2	29.45	-4.95	38.75	-17.75	60.00	82.00	5.10	158.70
A. arguta var. *giraldii*	Octaploid	SBX	1232.00	1860.2	31.20	-3.10	41.70	-16.10	60.00	77.00	5.10	125.20
A. arguta var. *giraldii*	Decaploid	SBX	1291.00	2120.00	30.85	-3.45	41.35	-16.45	60.00	77.00	5.10	125.20
A. arguta var. *giraldii*	Decaploid	SBX	1350.00	2120.00	30.49	-3.81	40.99	-16.81	60.00	77.00	5.10	125.20
A. arguta var. *giraldii*	Decaploid	SBX	1350.00	2120.00	30.49	-3.81	40.99	-16.81	60.00	77.00	5.10	125.20
A. arguta var. *giraldii*	Decaploid	SBX	1289.00	2120.00	30.86	-3.44	41.36	-16.44	60.00	77.00	5.10	125.20
A. arguta var. *giraldii*	Octaploid	HNT	1005.00	1800.00	30.48	-2.52	40.18	-12.52	63.00	81.00	13.60	184.80
A. arguta var. *arguta*	Tetraploid	HNT	1005.00	1800.00	30.48	-2.52	40.18	-12.52	63.00	81.00	13.60	184.80
A. arguta var. *arguta*	Tetraploid	HNT	1033.40	1800.00	30.31	-2.69	40.01	-12.69	63.00	81.00	13.60	184.80
A. arguta var. *arguta*	Tetraploid	HNT	1061.80	1800.00	30.14	-2.86	39.84	-12.86	63.00	81.00	13.60	184.80
A. arguta var. *arguta*	Tetraploid	HNT	1090.20	1800.00	29.97	-3.03	39.67	-13.03	63.00	81.00	13.60	184.80
A. arguta var. *arguta*	Tetraploid	HNT	1118.60	1800.00	29.80	-3.20	39.50	-13.20	63.00	81.00	13.60	184.80
A. arguta var. *arguta*	Tetraploid	HNT	1147.00	1800.00	29.63	-3.37	39.33	-13.37	63.00	81.00	13.60	184.80
A. arguta var. *arguta*	Tetraploid	HNT	1175.40	1800.00	29.46	-3.54	39.16	-13.54	63.00	81.00	13.60	184.80
A. arguta var. *arguta*	Tetraploid	HNT	1203.80	1800.00	29.29	-3.71	38.99	-13.71	63.00	81.00	13.60	184.80

A. arguta var. *arguta*	Tetraploid	HNT	1232.20	1800.00	29.12	-3.88	38.82	-13.88	63.00	81.00	13.60	184.80
A. arguta var. *arguta*	Tetraploid	HNT	1260.60	1800.00	28.95	-4.05	38.65	-14.05	63.00	81.00	13.60	184.80
A. arguta var. *arguta*	Tetraploid	HNT	1289.00	1800.00	28.78	-4.22	38.48	-14.22	63.00	81.00	13.60	184.80
A. arguta var. *arguta*	Tetraploid	HNT	1317.40	1800.00	28.61	-4.39	38.31	-14.39	63.00	81.00	13.60	184.80
A. arguta var. *arguta*	Tetraploid	HNT	1345.80	1800.00	28.44	-4.56	38.14	-14.56	63.00	81.00	13.60	184.80
A. arguta var. *arguta*	Tetraploid	HNT	1374.20	1800.00	28.26	-4.74	37.96	-14.74	63.00	81.00	13.60	184.80
A. arguta var. *arguta*	Tetraploid	HNT	1402.60	1800.00	28.09	-4.91	37.79	-14.91	63.00	81.00	13.60	184.80
A. arguta var. *arguta*	Tetraploid	HNT	1431.00	1800.00	27.92	-5.08	37.62	-15.08	63.00	81.00	13.60	184.80
A. arguta var. *giraldii*	Octaploid	HNS	1276.00	1800.00	28.85	-4.15	38.55	-14.15	63.00	81.00	13.60	184.80
A. arguta var. *giraldii*	Octaploid	HNS	1315.33	1800.00	28.62	-4.38	38.32	-14.38	63.00	81.00	13.60	184.80
A. arguta var. *giraldii*	Octaploid	HNS	1354.67	1800.00	28.38	-4.62	38.08	-14.62	63.00	81.00	13.60	184.80
A. arguta var. *giraldii*	Octaploid	HNS	1394.00	1800.00	28.15	-4.85	37.85	-14.85	63.00	81.00	13.60	184.80
A. arguta var. *giraldii*	Octaploid	HNS	1433.33	1800.00	27.91	-5.09	37.61	-15.09	63.00	81.00	13.60	184.80
A. arguta var. *giraldii*	Octaploid	HNS	1472.67	1800.00	27.67	-5.33	37.37	-15.33	63.00	81.00	13.60	184.80
A. arguta var. *giraldii*	Octaploid	HNS	1512.00	1800.00	27.44	-5.56	37.14	-15.56	63.00	81.00	13.60	184.80
A. arguta var. *arguta*	Tetraploid	HNS	1148.00	1800.00	29.62	-3.38	39.32	-13.38	63.00	81.00	13.60	184.80
A. arguta var. *arguta*	Tetraploid	HNS	1149.33	1800.00	29.61	-3.39	39.31	-13.39	63.00	81.00	13.60	184.80
A. arguta var. *arguta*	Tetraploid	HNS	1150.67	1800.00	29.61	-3.39	39.31	-13.39	63.00	81.00	13.60	184.80
A. arguta var. *arguta*	Tetraploid	HNS	1152.00	1800.00	29.60	-3.40	39.30	-13.40	63.00	81.00	13.60	184.80
A. arguta var. *arguta*	Tetraploid	HNL	835.00	1800.00	31.50	-1.50	41.20	-11.50	63.00	81.00	13.60	184.80
A. arguta var. *arguta*	Tetraploid	HNL	858.00	1800.00	31.36	-1.64	41.06	-11.64	63.00	81.00	13.60	184.80
A. arguta var. *arguta*	Tetraploid	HNL	881.00	1800.00	31.22	-1.78	40.92	-11.78	63.00	81.00	13.60	184.80
A. arguta var. *arguta*	Tetraploid	HNL	904.00	1800.00	31.09	-1.91	40.79	-11.91	63.00	81.00	13.60	184.80
A. arguta var. *arguta*	Tetraploid	HNL	927.00	1800.00	30.95	-2.05	40.65	-12.05	63.00	81.00	13.60	184.80
A. arguta var. *arguta*	Tetraploid	HNL	950.00	1800.00	30.81	-2.19	40.51	-12.19	63.00	81.00	13.60	184.80
A. arguta var. *arguta*	Tetraploid	HNL	973.00	1800.00	30.67	-2.33	40.37	-12.33	63.00	81.00	13.60	184.80
A. arguta var. *arguta*	Tetraploid	HNL	996.00	1800.00	30.53	-2.47	40.23	-12.47	63.00	81.00	13.60	184.80
A. arguta var. *arguta*	Tetraploid	HNL	1019.00	1800.00	30.40	-2.60	40.10	-12.60	63.00	81.00	13.60	184.80
A. arguta var. *arguta*	Tetraploid	HNL	1042.00	1800.00	30.26	-2.74	39.96	-12.74	63.00	81.00	13.60	184.80
A. arguta var. *arguta*	Tetraploid	HNL	1065.00	1800.00	30.12	-2.88	39.82	-12.88	63.00	81.00	13.60	184.80
A. arguta var. *arguta*	Tetraploid	HNL	1088.00	1800.00	29.98	-3.02	39.68	-13.02	63.00	81.00	13.60	184.80
A. arguta var. *arguta*	Tetraploid	HNL	1111.00	1800.00	29.84	-3.16	39.54	-13.16	63.00	81.00	13.60	184.80
A. arguta var. *arguta*	Tetraploid	HLS	1140.00	1800.00	29.67	-3.33	39.37	-13.33	63.00	81.00	13.60	184.80
A. arguta var. *arguta*	Tetraploid	HLS	1176.50	1800.00	29.45	-3.55	39.15	-13.55	63.00	81.00	13.60	184.80
A. arguta var. *arguta*	Tetraploid	HLS	1213.00	1800.00	29.23	-3.77	38.93	-13.77	63.00	81.00	13.60	184.80
A. arguta var. *arguta*	Tetraploid	HLS	1249.50	1800.00	29.01	-3.99	38.71	-13.99	63.00	81.00	13.60	184.80
A. arguta var. *arguta*	Tetraploid	HLS	1286.00	1800.00	28.79	-4.21	38.49	-14.21	63.00	81.00	13.60	184.80
A. arguta var. *arguta*	Tetraploid	HLS	1322.50	1800.00	28.58	-4.43	38.28	-14.43	63.00	81.00	13.60	184.80

A. arguta var. *arguta*	Tetraploid	HLS	1359.00	1800.00	28.36	-4.64	38.06	-14.64	63.00	81.00	13.60	184.80
A. melanandra	Tetraploid	GTM	1560.00	2100	29.40	-5.90	37.20	-17.60	61.00	78.00	3.30	91.80
A. melanandra	Tetraploid	GTM	1606.00	2100	29.12	-6.18	36.92	-17.88	61.00	78.00	3.30	91.80
A. arguta var. *arguta*	Tetraploid	GTM	1603.00	2100	29.14	-6.16	36.94	-17.86	61.00	78.00	3.30	91.80
A. melanandra	Tetraploid	GHM	1400.00	2100	29.40	-4.00	37.80	-16.40	67.00	85.00	5.60	133.70
A. melanandra	Tetraploid	GHM	1460.00	2100	29.04	-4.36	37.44	-16.76	67.00	85.00	5.60	133.70
A. melanandra	Tetraploid	GHM	1520.00	2100	28.68	-4.72	37.08	-17.12	67.00	85.00	5.60	133.70
A. arguta var. *giraldii*	Decaploid	GHM	1455.00	1726.4	29.07	-4.33	37.47	-16.73	67.00	85.00	5.60	133.70
A. arguta var. *giraldii*	Decaploid	GHM	1463.67	1726.4	29.02	-4.38	37.42	-16.78	67.00	85.00	5.60	133.70
A. arguta var. *giraldii*	Decaploid	GHM	1472.33	1726.4	28.97	-4.43	37.37	-16.83	67.00	85.00	5.60	133.70
A. arguta var. *giraldii*	Decaploid	GHM	1481.00	1726.4	28.91	-4.49	37.31	-16.89	67.00	85.00	5.60	133.70

Supplement Table 2 The gronomic trait and ploidy data of *A. arguta* var. *arguta* , *A. arguta* var. *giraldii* and *A. melananc*

Speceis	Ploidy levels	Leaf length (mm)	Leaf width (mm)	Petiole length (mm)	Flower diameter (mm)	Fruit length (mm)	Fruit greater diameter (mm)	Fruit Lesser diameter (mm)	Fruit weight (g)	Soluble solids content (%)
A. arguta var. *arguta*	Tetraploid	91.49	36.5	45.36	18	23.56	20.03	17.01	4.28	13.6
A. arguta var. *arguta*	Tetraploid	94.63	55.95	44.77	18.1	24.8	22.3	19.07	5.86	14.5
A. arguta var. *arguta*	Tetraploid	99.34	55.16	42.39	18.2	25.52	14.73	15.46	3.11	16.4
A. arguta var. *arguta*	Tetraploid	85.08	33.95	42.18	16.9	21.91	19.63	15.82	3.99	13.4
A. arguta var. *arguta*	Tetraploid	88	52.03	41.63	22.8	23.07	20.74	16.73	5.45	16.2
A. arguta var. *arguta*	Tetraploid	92.38	51.29	39.42	18.5	23.73	13.7	13.45	2.9	15.3
A. arguta var. *arguta*	Tetraploid	82.57	53.5	34.08	18.6	17.35	16.13	14.52	2.63	14.5
A. arguta var. *arguta*	Tetraploid	72.21	47.23	35.55	17.5	20.26	16.52	16.55	3.97	13.2
A. arguta var. *arguta*	Tetraploid	82.89	49.9	30.58	20.7	28.4	18.67	16.23	4.8	13.7
A. arguta var. *arguta*	Tetraploid	95.22	54.52	29.02	18.8	25.32	19.18	16.5	5.4	13.62
A. arguta var. *arguta*	Tetraploid	110.83	52.59	56.37	18.9	20.35	17.35	16.22	3.5	14.52
A. arguta var. *arguta*	Tetraploid	104.26	64.85	49.27	20.3	35.53	15.34	14.88	4.78	16.42
A. arguta var. *arguta*	Tetraploid	107.81	69.09	58.67	19.1	32.87	14.72	14.4	4.13	18.52
A. arguta var. *arguta*	Tetraploid	102.43	65.68	47.06	24.3	33.64	20.2	18.87	8.22	12.41
A. arguta var. *arguta*	Tetraploid	79.79	53.69	35.46	17.8	18.19	14.8	13.82	2.44	9.91
A. arguta var. *arguta*	Tetraploid	88	53.59	43.84	17.9	34.26	19.13	18.02	7.21	9.71
A. arguta var. *arguta*	Tetraploid	101.73	61.95	52.68	18	39.61	22.11	20.84	8.34	13.63
A. arguta var. *arguta*	Tetraploid	87.48	42.55	42.37	17.5	28.53	19.33	17.98	5	14.53
A. arguta var. *arguta*	Tetraploid	91.47	36.49	45.35	18.2	23.55	20.03	17.01	4.28	16.44
A. arguta var. *arguta*	Tetraploid	94.6	55.93	44.76	18.3	24.8	22.29	19.05	5.86	18.54
A. arguta var. *arguta*	Tetraploid	95.15	37.97	47.17	18.4	24.51	20.84	17.69	4.45	12.43
A. arguta var. *arguta*	Tetraploid	98.41	58.18	46.56	18.3	25.8	23.19	19.82	6.09	9.92
A. arguta var. *arguta*	Tetraploid	103.31	36.49	44.08	18.6	26.53	15.32	15.04	3.23	9.72
A. arguta var. *arguta*	Tetraploid	92.33	58.38	38.11	19.6	19.39	18.05	16.25	2.94	14.23
A. arguta var. *arguta*	Tetraploid	80.76	52.82	39.76	18.7	24.89	18.48	17.39	4.34	15.24
A. arguta var. *arguta*	Tetraploid	92.7	57.36	34.19	20.1	31.76	19.77	18.14	5.37	15.12
A. arguta var. *arguta*	Tetraploid	106.48	59.83	39.44	18.9	28.31	21.44	18.46	6.04	14.33
A. arguta var. *arguta*	Tetraploid	123.94	58.81	63.03	24.1	22.76	19.4	18.14	3.91	13.04
A. arguta var. *arguta*	Tetraploid	116.58	72.52	55.1	18.5	39.74	17.16	16.64	5.34	13.54
A. arguta var. *arguta*	Tetraploid	82.89	77.27	65.61	17.7	36.75	16.47	16.1	4.61	13.46

A. arguta var. *arguta*	Tetraploid	95.22	73.45	52.63	19.7	37.62	22.59	21.09	9.19	14.35
A. arguta var. *arguta*	Tetraploid	75.68	36.81	36.66	17.3	24.68	16.72	15.55	4.33	14.22
A. arguta var. *arguta*	Tetraploid	88.78	57.53	36.64	18	18.65	17.35	15.62	2.82	18.5
A. arguta var. *arguta*	Tetraploid	77.65	50.79	38.22	18.4	23.93	17.77	16.73	4.17	12.4
A. arguta var. *arguta*	Tetraploid	89.14	53.66	32.88	18.5	30.54	19.01	17.45	5.16	9.9
A. arguta var. *arguta*	Tetraploid	102.38	58.63	31.19	18.6	27.23	20.62	17.74	5.81	9.7
A. arguta var. *arguta*	Tetraploid	119.17	56.54	60.61	18.1	21.88	18.65	17.44	3.76	14.2
A. arguta var. *arguta*	Tetraploid	112.1	69.73	52.98	18.7	38.21	16.5	16	5.13	15.2
A. arguta var. *arguta*	Tetraploid	115.93	74.29	63.08	21.8	35.34	15.83	15.48	4.43	14.3
A. arguta var. *arguta*	Tetraploid	110.14	70.62	50.61	18.9	36.17	22.73	20.28	8.83	13.3
A. arguta var. *arguta*	Tetraploid	85.79	57.73	38.12	17.9	19.55	15.92	14.87	2.63	12.4
A. arguta var. *arguta*	Tetraploid	94.63	57.63	49.14	19.1	32.84	20.58	19.38	7.76	12.5
A. arguta var. *arguta*	Tetraploid	81.38	39.58	39.41	19.2	26.53	17.98	16.73	4.65	14.5
A. arguta var. *arguta*	Tetraploid	98.34	39.24	48.76	19.3	25.33	21.54	18.28	4.61	15.22
A. arguta var. *arguta*	Tetraploid	101.73	60.14	48.12	20.7	26.66	23.97	20.49	6.3	14.32
A. arguta var. *arguta*	Tetraploid	106.79	59.29	45.57	19.5	27.43	15.84	15.55	3.35	13.32
A. arguta var. *arguta*	Tetraploid	95.44	61.85	39.39	23.8	20.04	18.65	16.79	3.03	12.41
A. arguta var. *arguta*	Tetraploid	83.47	54.59	41.09	18.2	25.73	19.09	17.98	4.48	12.52
A. arguta var. *arguta*	Tetraploid	95.82	57.69	35.34	20.8	32.83	20.43	18.75	5.54	14.52
A. arguta var. *arguta*	Tetraploid	110.06	63.02	33.54	18.4	29.26	22.16	19.08	6.24	13.42
A. arguta var. *arguta*	Tetraploid	128.1	60.78	65.15	22.8	23.53	20.04	18.75	4.04	16.22
A. arguta var. *arguta*	Tetraploid	120.51	74.97	56.95	18.6	41.08	17.73	17.2	5.52	15.32
A. arguta var. *arguta*	Tetraploid	124.63	79.87	67.82	21.6	37.99	17.02	16.65	4.77	14.52
A. arguta var. *arguta*	Tetraploid	118.4	75.93	54.4	18.7	38.88	23.36	21.8	9.5	13.22
A. arguta var. *arguta*	Tetraploid	92.23	62.06	40.99	17.7	21.02	17.11	15.99	2.81	13.72
A. arguta var. *arguta*	Tetraploid	87.96	35.1	43.61	18.9	22.66	18.26	16.35	4.12	16.23
A. arguta var. *arguta*	Tetraploid	90.98	53.79	43.05	19	25.85	21.44	18.33	5.64	18.3
A. arguta var. *arguta*	Tetraploid	95.51	53.03	40.76	19.1	24.54	14.17	13.9	2.99	12.27
A. arguta var. *arguta*	Tetraploid	85.37	55.31	35.23	20.5	17.93	16.68	15.02	2.72	9.79
A. arguta var. *arguta*	Tetraploid	74.66	48.84	36.76	19.3	23.01	17.08	16.08	4.01	9.6
A. arguta var. *arguta*	Tetraploid	85.7	51.6	31.61	24.5	29.37	18.28	16.77	4.96	14.05
A. arguta var. *arguta*	Tetraploid	120.1	56.36	30	20.8	26.18	19.82	17.06	5.59	15.04
A. arguta var. *arguta*	Tetraploid	102.83	54.37	58.28	18.1	21.04	17.93	16.77	4.61	14.92
A. arguta var. *arguta*	Tetraploid	107.79	67.05	50.95	19.1	36.74	15.86	15.39	4.94	14.14
A. arguta var. *giraldii*	Octaploid	106.26	68.81	51.65	20	23.92	12.78	11.96	1.91	11.9

A. arguta var. *giraldii*	Octaploid	101.99	57.01	51.12	20.2	24.59	12.56	11.65	1.97	11.9
A. arguta var. *giraldii*	Octaploid	129.4	82.9	75.55	21.4	33.03	13.98	14.19	3.37	13
A. arguta var. *giraldii*	Octaploid	120.1	73.66	43.75	20.5	34.6	10.73	11.02	1.98	12.8
A. arguta var. *giraldii*	Octaploid	102.83	59.85	53.76	18.6	36.82	13.09	12.95	2.91	12.8
A. arguta var. *giraldii*	Octaploid	102.1	66.6	37.42	18.6	24.35	13.3	13.28	1.71	12.1
A. arguta var. *giraldii*	Octaploid	111.67	93.26	66.3	20.8	26.27	12.43	12.55	1.64	15
A. arguta var. *giraldii*	Octaploid	118.87	76.34	64.94	19.3	35.52	14.27	14.23	3.76	15.1
A. arguta var. *giraldii*	Octaploid	112.94	72.57	52.1	21.2	36.35	19.58	18.64	7.49	14.8
A. arguta var. *giraldii*	Octaploid	86.73	34.67	43.17	20.4	21.89	16.7	14.45	3.36	14.8
A. arguta var. *giraldii*	Octaploid	89.71	53.15	42.61	20.6	23.05	18.59	16.2	4.59	14.8
A. arguta var. *giraldii*	Octaploid	94.18	52.39	43.34	20.8	23.71	12.28	13.3	2.44	15.4
A. arguta var. *giraldii*	Octaploid	84.17	54.65	34.88	20.9	17.33	14.46	13.27	2.22	15.1
A. arguta var. *giraldii*	Octaploid	101.68	54.65	36.38	18.8	22.24	14.81	14.22	3.27	13
A. arguta var. *giraldii*	Octaploid	129.01	48.24	55.54	18.9	25.71	13.74	12.85	2.05	12.8
A. arguta var. *giraldii*	Octaploid	109.67	73.99	54.97	21.2	26.44	13.5	12.53	2.12	12.3
A. arguta var. *giraldii*	Octaploid	139.14	89.14	81.23	19.7	35.52	15.04	15.25	3.62	12.1
A. arguta var. *giraldii*	Decaploid	129.14	79.2	47.03	18.6	37.2	11.54	11.86	2.14	15
A. arguta var. *giraldii*	Decaploid	110.56	64.35	55.81	18.6	39.6	14.08	12.93	3.12	15.1
A. arguta var. *giraldii*	Decaploid	109.77	71.61	40.23	21.1	36.18	13.23	13.2	1.84	14.8
A. arguta var. *giraldii*	Decaploid	120.07	100.27	71.3	18.3	28.26	13.36	13.48	4.76	10.2
A. arguta var. *giraldii*	Decaploid	105.93	68.61	51.5	22.2	23.85	12.75	11.92	1.9	12
A. arguta var. *giraldii*	Decaploid	101.68	56.84	50.98	19.2	34.52	12.52	11.61	1.96	10
A. arguta var. *giraldii*	Decaploid	129.01	82.65	75.32	19.8	32.94	13.94	14.15	3.36	10
A. arguta var. *giraldii*	Decaploid	119.73	73.43	43.62	22.6	34.5	10.7	11.99	3.98	9.7
A. arguta var. *giraldii*	Decaploid	102.51	59.68	53.6	20.1	36.72	13.25	12.91	2.9	9
A. arguta var. *giraldii*	Decaploid	101.79	66.4	37.31	18.8	24.28	12.27	12.23	3.7	12.1
A. arguta var. *giraldii*	Decaploid	111.33	92.98	66.1	18.9	26.2	12.38	12.51	1.64	12.4
A. melanandra	Tetraploid	79.14	37.27	31.22	18.6	35.88	22.88	19.17	8.55	12.5
A. melanandra	Tetraploid	92.22	46.58	28.08	18.6	27.08	18.3	16.58	5.31	13
A. melanandra	Tetraploid	86.75	36.37	29.8	18.7	34.56	17.05	15.42	4.94	13.8
A. melanandra	Tetraploid	102.12	73.25	43.15	17.9	36.83	21.6	19.99	7.94	17.6
A. melanandra	Tetraploid	96.54	45.21	36.91	16.6	29.3	19.25	18.61	4.24	14.3
A. melanandra	Tetraploid	75.05	38.68	43.95	17.1	23.97	24.31	22.62	7.36	9.8
A. melanandra	Tetraploid	84.05	42.7	54.27	19.1	24.48	26.09	23.4	8.27	10.4
A. melanandra	Tetraploid	67.46	32.45	18.35	19.1	30.93	21.41	20.18	7.8	15.3
A. melanandra	Tetraploid	80.68	43.4	36.81	16.8	28.15	20.48	19.18	6.54	13.2

A. melanandra	Tetraploid	94.97	39.58	36.91	19.4	29.03	20.68	19.14	6.64	14.6
A. melanandra	Tetraploid	114.94	50.33	57.53	17.7	26.12	24.3	22.03	7.91	13.2
A. melanandra	Tetraploid	109.44	51.34	44.61	19.6	27.86	19.28	19.23	4.9	12.5
A. melanandra	Tetraploid	73.6	34.65	29.04	19.3	33.36	21.27	18.82	7.95	13.06
A. melanandra	Tetraploid	85.76	43.32	26.12	22.4	25.18	17.01	15.42	4.95	13.59
A. melanandra	Tetraploid	80.68	33.82	27.72	19.5	32.13	15.85	14.34	4.59	14.42
A. melanandra	Tetraploid	94.97	68.12	40.13	21.6	34.25	20.08	18.59	7.38	18.39
A. melanandra	Tetraploid	89.79	42.04	34.32	19.4	29.25	17.9	18.31	3.95	14.94
A. melanandra	Tetraploid	69.8	35.97	51.57	18.8	22.3	22.6	21.03	6.85	10.24
A. melanandra	Tetraploid	78.17	39.71	50.48	21.7	22.76	24.26	21.76	7.7	10.87
A. melanandra	Tetraploid	79.94	41.19	36.15	17.3	25.54	25.88	24.09	7.83	15.99
A. melanandra	Tetraploid	89.51	45.47	47.81	20.3	29.07	26.79	24.92	8.82	13.79
A. melanandra	Tetraploid	71.84	34.56	19.55	19.2	32.95	22.8	21.49	8.31	15.26
A. melanandra	Tetraploid	85.37	46.23	39.2	19.8	29.98	21.81	20.42	6.96	13.79
A. melanandra	Tetraploid	89.09	42.15	39.3	19.3	31.91	22.03	20.38	7.07	13.06
A. melanandra	Tetraploid	122.41	53.6	61.27	20	27.83	25.87	23.46	8.42	13.2
A. melanandra	Tetraploid	116.54	54.67	47.5	23.1	29.66	20.53	17.28	5.22	15.5
A. melanandra	Tetraploid	83.19	42.02	25.33	18.3	24.42	16.51	14.96	4.8	13.18

ra

Ascorbic acid content(mg/100g fresh weight)	Total sugar content (g/100g fresh weight)	Total acid (g/100g fresh weight)	Total amino acid content (g/100g fresh weight)	Asparagine ((g/100g fresh weight))	Threonine (g/100g fresh weight)	Serine (g/100g fresh weight)	Glutamic acid (g/100g fresh weight)	Proline (g/100g fresh weight)	Glycine (g/100g fresh weight)	Alanine (g/100g fresh weight)	Cystine (g/100g fresh weight)
26.99	8.16	0.78	0.7539	0.094	0.0434	0.0408	0.0984	0.068	0.0466	0.0445	0.0093
52.3	8.35	1.07	0.86	0.0971	0.0457	0.0428	0.139	0.0768	0.0515	0.055	0.0113
52.58	8.62	1.21	1.1449	0.1389	0.0492	0.0550	0.1653	0.088	0.0587	0.0707	0.0146
25.1	7.59	0.72	0.701	0.0874	0.0404	0.0379	0.0915	0.0633	0.0433	0.0414	0.0087
48.63	7.78	0.99	0.7998	0.0903	0.0425	0.0398	0.1293	0.0714	0.0479	0.0512	0.0105
48.9	7.09	1.12	1.0647	0.1292	0.0457	0.0511	0.1537	0.0819	0.0546	0.0657	0.0135
45.28	8.17	0.83	0.766	0.0952	0.0467	0.0425	0.0986	0.0643	0.0511	0.0458	0.0132
16.43	5.1	1.3	0.7379	0.0832	0.0382	0.0376	0.1144	0.0693	0.0445	0.0456	0.0102
51.99	7.53	1.1	0.5493	0.0629	0.0399	0.0327	0.0761	0.0557	0.0409	0.0337	0.0091
81.18	8.86	1.23	0.6593	0.0767	0.038	0.0362	0.0929	0.0595	0.0409	0.0416	0.0084
56.8	10.59	0.9	1.014	0.1129	0.056	0.0530	0.1414	0.0831	0.0602	0.0702	0.017
54.62	10.18	0.86	0.9749	0.1085	0.0538	0.0510	0.1359	0.0799	0.0579	0.0675	0.0164
23.01	6.95	1.27	0.977	0.1083	0.047	0.0505	0.1742	0.0888	0.0528	0.0712	0.0167
54.6	7.67	1.15	0.9868	0.1175	0.0475	0.0695	0.1552	0.0821	0.0535	0.0612	0.0133
68.19	7.17	1.18	1.22	0.1496	0.0546	0.0565	0.1763	0.0905	0.0597	0.0699	0.0155
52.06	8.77	1.39	1.0321	0.1236	0.0522	0.0542	0.1741	0.092	0.058	0.0683	0.0152
60.18	10.14	1.6	1.193	0.1429	0.0604	0.0627	0.2012	0.1062	0.0671	0.0789	0.0176
59.79	9.88	2.11	1.0214	0.1055	0.0506	0.0532	0.1634	0.1006	0.0581	0.0604	0.0142
26.98	8.16	0.78	0.7537	0.094	0.0434	0.0408	0.0983	0.068	0.0466	0.0445	0.0093
52.29	8.35	1.07	0.8598	0.097	0.0457	0.0427	0.139	0.0768	0.0515	0.055	0.0112
28.07	8.48	0.81	0.784	0.0978	0.0452	0.0424	0.1022	0.0707	0.0485	0.0463	0.0097
54.39	8.69	1.11	0.8944	0.1008	0.0475	0.0445	0.1446	0.0799	0.0535	0.0572	0.0117
54.68	7.93	1.26	1.1907	0.1445	0.0512	0.0572	0.1718	0.0916	0.061	0.0735	0.0151
61.81	9.13	0.93	0.8566	0.1064	0.0522	0.0475	0.1102	0.0719	0.0571	0.0513	0.0148
18.37	5.71	1.34	0.8252	0.0931	0.0428	0.0420	0.1279	0.0776	0.0497	0.051	0.0114
58.15	8.41	1.19	0.7142	0.0704	0.0446	0.0365	0.0851	0.0623	0.0458	0.0377	0.0102
90.79	9.91	1.38	0.7373	0.0857	0.0425	0.0405	0.1038	0.0666	0.0457	0.0466	0.0094
63.52	11.83	1	1.1339	0.1262	0.0626	0.0593	0.1581	0.0929	0.0674	0.0785	0.019
61.07	11.39	0.96	1.0903	0.1214	0.0602	0.0570	0.152	0.0894	0.0648	0.0755	0.0183
25.73	7.77	1.42	1.0927	0.1212	0.0526	0.0565	0.1948	0.0994	0.0591	0.0797	0.0187

61.05	8.57	1.29	1.1035	0.1314	0.0531	0.0777	0.1734	0.0918	0.0599	0.0685	0.0149
51.73	8.55	1.82	0.8836	0.0913	0.0438	0.0460	0.1413	0.0871	0.0502	0.0522	0.0123
59.44	8.78	0.89	0.8237	0.1023	0.0502	0.0457	0.1059	0.0691	0.0549	0.0493	0.0112
17.66	5.49	1.29	0.7934	0.0895	0.0411	0.0404	0.123	0.0746	0.0478	0.0491	0.0109
55.91	8.1	1.15	0.5906	0.0677	0.0429	0.0350	0.0818	0.0599	0.044	0.0362	0.0098
87.29	9.53	1.33	0.7089	0.0824	0.0408	0.0390	0.0999	0.064	0.044	0.0448	0.009
61.07	11.39	0.96	1.0903	0.1214	0.0602	0.0570	0.152	0.0894	0.0648	0.0755	0.0183
58.73	10.95	0.93	1.0483	0.1167	0.0579	0.0548	0.1462	0.0859	0.0623	0.0726	0.0176
24.75	7.47	1.37	1.0506	0.1165	0.0506	0.0543	0.1873	0.0955	0.0568	0.0766	0.018
58.71	8.25	1.24	1.0611	0.1263	0.0511	0.0747	0.1667	0.0883	0.0576	0.0658	0.0143
63.32	7.71	1.26	1.3118	0.1609	0.0587	0.0608	0.1896	0.0973	0.0642	0.0752	0.0166
55.97	9.43	1.49	1.1098	0.133	0.0562	0.0583	0.1872	0.0989	0.0624	0.0734	0.0164
55.62	9.19	1.96	0.9501	0.0982	0.0471	0.0495	0.152	0.0937	0.054	0.0561	0.0132
29.01	8.77	0.84	0.8104	0.1009	0.0467	0.0439	0.1056	0.0731	0.0501	0.0478	0.01
56.22	8.98	1.15	0.9245	0.1042	0.0491	0.0460	0.1495	0.0826	0.0553	0.0591	0.0121
56.53	8.2	1.3	1.2308	0.1493	0.0529	0.0591	0.1776	0.0947	0.0631	0.076	0.0156
63.89	9.44	0.96	0.8855	0.11	0.054	0.0492	0.1139	0.0743	0.059	0.053	0.0153
18.99	5.9	1.38	0.8529	0.0962	0.0442	0.0434	0.1322	0.0802	0.0514	0.0528	0.0118
60.11	8.7	1.23	0.6348	0.0727	0.0461	0.0377	0.088	0.0644	0.0473	0.039	0.0105
93.84	10.25	1.43	0.7622	0.0886	0.0439	0.0419	0.1073	0.0688	0.0473	0.0482	0.0097
65.65	12.23	1.04	1.172	0.1305	0.0647	0.0613	0.1635	0.0961	0.0697	0.0812	0.0197
63.14	11.76	1	1.127	0.1255	0.0622	0.0589	0.1572	0.0924	0.067	0.078	0.0189
26.6	8.04	1.47	1.1294	0.1252	0.0544	0.0584	0.2014	0.1026	0.0611	0.0823	0.0194
63.11	8.86	1.33	1.1407	0.1358	0.0549	0.0803	0.1793	0.0949	0.0619	0.0708	0.0154
78.81	8.29	1.36	1.4102	0.1729	0.0631	0.0654	0.2039	0.1045	0.069	0.0809	0.0179
25.95	7.85	0.75	0.7248	0.0904	0.0417	0.0392	0.0946	0.0654	0.0448	0.0428	0.0089
50.28	8.04	1.03	0.8269	0.0933	0.0439	0.0411	0.1337	0.0739	0.0495	0.0529	0.0108
50.56	7.33	1.16	1.1008	0.1335	0.0473	0.0529	0.1589	0.0846	0.0564	0.0679	0.014
57.15	8.44	0.86	0.792	0.0984	0.0483	0.0440	0.1018	0.0665	0.0528	0.0474	0.0137
16.99	5.28	1.24	0.7629	0.086	0.0395	0.0388	0.1182	0.0717	0.046	0.0472	0.0105
53.76	7.79	1.1	0.5678	0.065	0.0413	0.0337	0.0787	0.0576	0.0423	0.0348	0.0094
83.93	9.16	1.28	0.6817	0.0793	0.0392	0.0375	0.0961	0.0616	0.0423	0.0431	0.0087
58.72	10.95	0.93	1.0483	0.1167	0.0579	0.0548	0.1462	0.0859	0.0623	0.0726	0.0176
56.47	10.52	0.89	1.008	0.1122	0.0557	0.0527	0.1405	0.0826	0.0599	0.0698	0.0169
142.16	5	0.9	1.5292	0.1833	0.0748	0.0679	0.2417	0.121	0.0844	0.0847	0.0223

102.66	5.38	0.9	1.7628	0.2606	0.0819	0.0705	0.2575	0.1236	0.0842	0.0912	0.0225
56.95	6.79	0.88	2.0423	0.4264	0.0962	0.0836	0.3143	0.128	0.0872	0.126	0.0263
95.55	6.06	0.97	1.8731	0.2265	0.072	0.0672	0.2084	0.0999	0.07	0.0847	0.0182
86.59	6.42	0.92	1.84914	0.326	0.0868	0.0728	0.2599	0.1219	0.0824	0.0969	0.0231
115.29	5.62	0.54	1.473	0.1814	0.0765	0.0650	0.2188	0.1132	0.0857	0.0832	0.0199
116.28	7.33	0.85	1.5793	0.189	0.075	0.0666	0.249	0.1224	0.084	0.0833	0.0214
25.23	10.47	1.24	1.2347	0.1353	0.0544	0.0557	0.1966	0.0966	0.0583	0.0815	0.0188
59.85	6.87	1.12	1.0945	0.1467	0.0549	0.0766	0.175	0.0893	0.0591	0.0701	0.0149
35.43	6.81	0.65	1.1894	0.1008	0.0432	0.0387	0.0954	0.0636	0.0442	0.0438	0.009
49.29	6.81	0.89	1.1079	0.1041	0.0454	0.0406	0.1349	0.0718	0.0488	0.0541	0.0109
49.56	11.29	1.01	1.2867	0.1491	0.0489	0.0522	0.1604	0.0823	0.0557	0.0695	0.0141
56.01	8.51	0.75	1.0113	0.1098	0.0499	0.0434	0.1028	0.0647	0.0521	0.0485	0.0137
16.65	6.49	1.07	1.1228	0.096	0.0409	0.0384	0.1193	0.0697	0.0454	0.0483	0.0106
152.85	6.03	0.97	1.8365	0.1971	0.0804	0.0730	0.2599	0.1301	0.0908	0.0911	0.024
110.38	5.97	0.96	1.8869	0.2802	0.078	0.0748	0.2769	0.1729	0.0906	0.088	0.0642
61.25	5.64	0.95	2.3555	0.4585	0.1034	0.0899	0.338	0.1376	0.0938	0.1355	0.0283
102.74	7.4	1.04	1.9709	0.2435	0.0775	0.0722	0.2241	0.1075	0.0752	0.0911	0.0196
93.11	10.5	0.99	1.9786	0.3506	0.0933	0.0783	0.2795	0.131	0.0886	0.1041	0.0248
123.97	7.04	0.58	1.5216	0.195	0.0822	0.0699	0.2352	0.1217	0.0922	0.0894	0.0214
125.03	4.99	0.91	1.6356	0.2032	0.0807	0.0716	0.2678	0.1316	0.0903	0.0895	0.023
141.73	5.54	0.9	1.5737	0.1828	0.0746	0.0677	0.241	0.1206	0.0842	0.0845	0.0222
102.34	3.31	0.89	1.8358	0.2598	0.0816	0.0703	0.2567	0.1232	0.084	0.0909	0.0225
56.78	4.63	0.88	2.1328	0.4251	0.0959	0.0834	0.3133	0.1276	0.087	0.1257	0.0262
95.25	3.07	0.97	1.9	0.2259	0.0718	0.0670	0.2078	0.0997	0.0698	0.0845	0.0182
86.33	3.06	0.92	1.9834	0.325	0.0865	0.0726	0.2591	0.1215	0.0822	0.0966	0.023
114.94	5.55	0.54	1.3871	0.1808	0.0762	0.0648	0.2181	0.1128	0.0855	0.0829	0.0198
115.93	6.02	0.84	1.5932	0.1884	0.0748	0.0664	0.2483	0.122	0.0838	0.083	0.0214
76.86	7.82	1.31	0.9289	0.099	0.0474	0.0487	0.1635	0.0835	0.053	0.0542	0.0139
80.21	7.87	1.08	1.0247	0.108	0.0519	0.0531	0.1762	0.0953	0.0572	0.0595	0.0153
80.18	8.65	0.78	1.0187	0.107	0.0527	0.0515	0.1643	0.0967	0.0575	0.0594	0.0145
73.87	10.43	0.91	1.0403	0.1095	0.0533	0.0532	0.1721	0.0984	0.0586	0.0607	0.015
78.75	8.76	1.27	0.8806	0.0925	0.0438	0.0464	0.1625	0.0774	0.0492	0.0524	0.0135
58.36	6.73	0.82	1.0377	0.1096	0.052	0.0550	0.1886	0.0936	0.0572	0.0599	0.0165
102.49	7.24	0.75	1.0057	0.1053	0.0525	0.0506	0.1603	0.0946	0.0566	0.0584	0.0146
62.32	9.45	1.33	0.7586	0.0779	0.0376	0.0400	0.1396	0.0641	0.0409	0.0478	0.0115
99.3	8.38	1.3	0.9761	0.1029	0.0488	0.0516	0.177	0.0879	0.0537	0.0562	0.0155

75.51	9.01	1.37	1.0286	0.1093	0.0511	0.0540	0.1931	0.0908	0.0598	0.0604	0.0152
55.34	8.26	1.25	0.7765	0.0839	0.0421	0.0401	0.1193	0.0714	0.0452	0.0457	0.0108
98.81	7.69	1.06	0.9252	0.0983	0.0487	0.0480	0.1502	0.0892	0.0536	0.0553	0.0114
71.49	7.93	1.22	0.8639	0.092	0.0441	0.0452	0.1521	0.0777	0.0493	0.0504	0.0129
74.6	8.38	1	0.953	0.1004	0.0483	0.0494	0.1639	0.0887	0.0532	0.0554	0.0142
74.57	8.87	0.73	0.9474	0.0996	0.049	0.0479	0.1528	0.09	0.0535	0.0553	0.0135
68.7	10.72	0.84	0.9675	0.1016	0.0495	0.0491	0.1575	0.0925	0.0547	0.0565	0.0135
73.24	9.1	1.19	0.8189	0.086	0.0407	0.0431	0.1511	0.0719	0.0457	0.0487	0.0125
54.28	8.03	0.77	0.9652	0.1018	0.0495	0.0494	0.1601	0.0915	0.0545	0.0564	0.0139
95.32	7.49	0.7	0.9353	0.098	0.0488	0.0471	0.1491	0.0879	0.0526	0.0543	0.0136
62.16	10.06	0.88	1.1052	0.1164	0.0567	0.0562	0.1802	0.1058	0.0626	0.0648	0.0154
109.16	8.6	0.8	1.0711	0.1122	0.0559	0.0539	0.1706	0.1006	0.0603	0.0622	0.0155
77.02	9.3	1.42	0.8079	0.083	0.0401	0.0426	0.1487	0.0683	0.0436	0.0509	0.0122
105.76	8.51	1.38	1.0395	0.1093	0.0532	0.0528	0.1692	0.0995	0.0588	0.0608	0.0145
80.42	8	1.46	1.0955	0.1164	0.0544	0.0575	0.2057	0.0967	0.0637	0.0643	0.0162
58.93	8.32	1.33	0.827	0.0894	0.0448	0.0427	0.1271	0.0768	0.0481	0.0487	0.0115
105.23	9.79	1.13	0.9854	0.1046	0.0519	0.0511	0.1599	0.095	0.0571	0.0589	0.0121
72.37	8.23	0.97	0.9243	0.0975	0.0468	0.0479	0.159	0.086	0.0516	0.0517	0.0138

Valine (g/100g fresh weight)	Methionine (g/100g fresh weight)	Isoleucine (g/100g fresh weight)	Leucine (g/100g fresh weight)	Tyrosine (g/100g fresh weight)	Phenylalanine (g/100g fresh	Lysine (g/100g fresh weight)	Histidine (g/100g fresh weight)	Arginine (g/100g fresh weight)	Total Anthocyanin (mg/100g fresh weight)
0.0454	0.005	0.0337	0.0558	0.0328	0.0264	0.0727	0.0177	0.0357	
0.0504	0.0052	0.0568	0.039	0.026	0.0311	0.0777	0.0188	0.0542	
0.0576	0.0054	0.0638	0.0461	0.0298	0.0361	0.0988	0.0257	0.056	
0.0422	0.0047	0.0314	0.0519	0.0305	0.0246	0.0676	0.0164	0.0333	
0.0468	0.0048	0.0528	0.0363	0.0242	0.0289	0.0722	0.0175	0.0504	
0.0536	0.005	0.0593	0.0428	0.0277	0.0335	0.0919	0.0239	0.0521	
0.0455	0.005	0.0487	0.0395	0.0362	0.0266	0.071	0.0167	0.0356	
0.0422	0.0033	0.0497	0.0338	0.0235	0.0287	0.0639	0.0173	0.0473	
0.0368	0.004	0.0424	0.0312	0.0255	0.0237	0.0539	0.013	0.0335	
0.0395	0.0047	0.0456	0.0318	0.0158	0.0257	0.0648	0.0158	0.0361	
0.059	0.0032	0.06	0.0494	0.0892	0.0297	0.0807	0.022	0.0475	
0.0567	0.0031	0.0577	0.0475	0.0858	0.0285	0.0776	0.0212	0.0457	
0.0521	0.0042	0.0612	0.0404	0.0269	0.0292	0.0848	0.0235	0.0651	
0.0529	0.0053	0.059	0.0415	0.0284	0.0334	0.088	0.0228	0.0528	
0.0596	0.0054	0.0647	0.0474	0.1706	0.0364	0.1007	0.0269	0.0617	
0.0571	0.0061	0.0648	0.0447	0.0285	0.038	0.0992	0.0261	0.0511	
0.066	0.0071	0.075	0.0517	0.0329	0.044	0.1146	0.0301	0.0591	
0.0585	0.0045	0.0755	0.0454	0.0255	0.0365	0.0925	0.027	0.0717	
0.0454	0.005	0.0337	0.0558	0.0328	0.0264	0.0727	0.0177	0.0357	
0.0504	0.0052	0.0568	0.039	0.026	0.0311	0.0777	0.0188	0.0542	
0.0472	0.0052	0.035	0.0581	0.034	0.0275	0.0756	0.0184	0.0372	
0.0524	0.0054	0.0591	0.0406	0.0271	0.0323	0.0808	0.0196	0.0564	
0.06	0.0056	0.0663	0.0479	0.031	0.0375	0.1027	0.0267	0.0583	
0.0509	0.0056	0.0545	0.0442	0.0404	0.0298	0.0794	0.0187	0.0399	
0.0472	0.0037	0.0555	0.0378	0.0263	0.0321	0.0715	0.0194	0.053	
0.0412	0.0045	0.0474	0.0348	0.0285	0.0265	0.0603	0.0145	0.0375	
0.0441	0.0052	0.0511	0.0354	0.0177	0.0287	0.0725	0.0176	0.0404	
0.066	0.0036	0.0671	0.0553	0.0998	0.0332	0.0903	0.0246	0.0532	
0.0635	0.0034	0.0645	0.0532	0.0959	0.0319	0.0868	0.0237	0.0511	
0.0583	0.0047	0.0685	0.0452	0.0301	0.0327	0.0948	0.0262	0.0728	

0.0591	0.0059	0.066	0.0464	0.0317	0.0373	0.0985	0.0255	0.059	
0.0506	0.0039	0.0653	0.0392	0.022	0.0317	0.08	0.0234	0.062	
0.0489	0.005	0.0524	0.0325	0.0389	0.0286	0.0763	0.018	0.0383	
0.0454	0.0036	0.0534	0.0364	0.0253	0.0309	0.0688	0.0186	0.0509	
0.0396	0.0043	0.0455	0.0334	0.0274	0.0255	0.058	0.0139	0.036	
0.0424	0.005	0.0491	0.0341	0.017	0.0276	0.0697	0.017	0.0388	
0.0635	0.0034	0.0645	0.0532	0.0959	0.0319	0.0868	0.0237	0.0511	
0.061	0.0033	0.062	0.0511	0.0922	0.0307	0.0835	0.0227	0.0492	
0.0561	0.0045	0.0658	0.0435	0.0289	0.0314	0.0912	0.0252	0.07	
0.0569	0.0057	0.0634	0.0446	0.0305	0.0359	0.0947	0.0245	0.0568	
0.0641	0.0058	0.0696	0.051	0.1834	0.0391	0.1083	0.029	0.0664	
0.0614	0.0066	0.0697	0.0481	0.0306	0.0409	0.1066	0.028	0.0549	
0.0544	0.0042	0.0702	0.0422	0.0237	0.0339	0.086	0.0251	0.0667	
0.0488	0.0054	0.0362	0.06	0.0352	0.0284	0.0781	0.019	0.0384	
0.0542	0.0056	0.0611	0.0419	0.028	0.0333	0.0835	0.0202	0.0583	
0.062	0.0058	0.0686	0.0495	0.032	0.0388	0.1062	0.0276	0.0602	
0.0526	0.0058	0.0563	0.0457	0.0418	0.0308	0.0821	0.0193	0.0412	
0.0488	0.0039	0.0574	0.0391	0.0272	0.0332	0.0739	0.02	0.0547	
0.0425	0.0046	0.049	0.036	0.0294	0.0274	0.0623	0.015	0.0387	
0.0456	0.0054	0.0528	0.0366	0.0183	0.0297	0.0749	0.0182	0.0418	
0.0682	0.0037	0.0694	0.0572	0.103	0.0342	0.0933	0.0254	0.055	3.05
0.0656	0.0035	0.0667	0.055	0.0991	0.033	0.0897	0.0245	0.0529	0.98
0.0603	0.0048	0.0708	0.0468	0.0311	0.0337	0.098	0.0271	0.0753	0.27
0.0611	0.0061	0.0682	0.048	0.0328	0.0386	0.1017	0.0263	0.061	0.76
0.0689	0.0062	0.0749	0.0548	0.1972	0.0421	0.1164	0.0311	0.0713	0.41
0.0436	0.0048	0.0325	0.0537	0.0316	0.0254	0.0699	0.017	0.0344	3.71
0.0484	0.005	0.0546	0.0375	0.025	0.0299	0.0747	0.0181	0.0521	0.71
0.0554	0.0052	0.0613	0.0443	0.0286	0.0347	0.095	0.0247	0.0539	2.65
0.0471	0.0052	0.0504	0.0409	0.0374	0.0275	0.0734	0.0173	0.0368	0.71
0.0436	0.0035	0.0513	0.035	0.0243	0.0297	0.0661	0.0179	0.049	0.68
0.038	0.0041	0.0438	0.0323	0.0263	0.0245	0.0558	0.0134	0.0346	1.71
0.0408	0.0048	0.0472	0.0329	0.0163	0.0266	0.067	0.0163	0.0373	2.30
0.061	0.0033	0.062	0.0511	0.0922	0.0307	0.0835	0.0227	0.0492	2.58
0.0587	0.0032	0.0596	0.0491	0.0887	0.0295	0.0803	0.0219	0.0473	2.03
0.0817	0.0048	0.0924	0.0643	0.0735	0.0495	0.0832	0.0328	0.1066	

0.0831	0.0073	0.0909	0.0649	0.1545	0.0497	0.0894	0.0332	0.1132	
0.091	0.0078	0.0959	0.0699	0.2144	0.0562	0.0959	0.0406	0.1295	
0.0715	0.005	0.0734	0.0574	0.4594	0.0397	0.0877	0.0321	0.0858	
0.0828	0.0076	0.0866	0.0642	0.2415	0.0514	0.0901	0.0331	0.1095	
0.0804	0.008	0.066	0.0843	0.0623	0.0562	0.086	0.0289	0.0728	
0.0814	0.0068	0.093	0.0641	0.0639	0.0469	0.0867	0.0326	0.1141	
0.0581	0.0044	0.0672	0.0449	0.0381	0.0322	0.0703	0.0262	0.0747	
0.0589	0.0055	0.0647	0.046	0.0402	0.0369	0.073	0.0255	0.0605	
0.0435	0.0045	0.0319	0.0532	0.04	0.0251	0.0518	0.017	0.0352	
0.0482	0.0046	0.0536	0.0372	0.0318	0.0295	0.0553	0.0181	0.0535	
0.0552	0.0049	0.0602	0.0439	0.0363	0.0342	0.0704	0.0247	0.0552	
0.0469	0.0048	0.0494	0.0405	0.0475	0.0272	0.0544	0.0173	0.0378	
0.0435	0.0032	0.0504	0.0347	0.0309	0.0293	0.049	0.0179	0.0502	
0.0879	0.0052	0.0994	0.0692	0.079	0.0532	0.0895	0.0352	0.1147	7.23
0.0893	0.0076	0.0967	0.0695	0.1662	0.0535	0.0962	0.0356	0.1217	7.33
0.0978	0.0084	0.103	0.0752	0.2306	0.0604	0.103	0.0436	0.1393	7.09
0.0769	0.0054	0.0789	0.0617	0.494	0.0427	0.0943	0.0344	0.0922	6.55
0.089	0.0082	0.0931	0.0691	0.2597	0.0553	0.0969	0.0355	0.1177	3.46
0.0865	0.0086	0.071	0.0907	0.067	0.0604	0.0925	0.0311	0.0783	3.23
0.0875	0.0073	0.0999	0.069	0.0687	0.0504	0.0932	0.0349	0.1227	3.07
0.0815	0.0048	0.0921	0.0641	0.0732	0.0493	0.083	0.0327	0.1063	7.84
0.0828	0.0073	0.0906	0.0648	0.1541	0.0496	0.0892	0.0331	0.1128	8.71
0.0907	0.0078	0.0956	0.0697	0.2138	0.056	0.0956	0.0405	0.1291	
0.0713	0.005	0.0731	0.0572	0.458	0.0396	0.0875	0.032	0.0855	
0.0825	0.0076	0.0863	0.064	0.2407	0.0513	0.0898	0.033	0.1091	
0.0802	0.008	0.0658	0.0841	0.0621	0.056	0.0857	0.0288	0.0726	
0.0811	0.0068	0.0927	0.0639	0.0637	0.0467	0.0864	0.0325	0.1138	
0.0507	0.0062	0.0611	0.0407	0.0333	0.0317	0.0583	0.023	0.0644	8.61
0.0566	0.0053	0.0698	0.0447	0.0311	0.0357	0.0687	0.0252	0.0736	9.58
0.0577	0.0047	0.0779	0.0459	0.0243	0.0374	0.0699	0.0253	0.0745	7.64
0.0586	0.0051	0.0749	0.0464	0.0271	0.0376	0.0708	0.0257	0.0761	8.52
0.0468	0.0041	0.0569	0.0372	0.0357	0.0304	0.0543	0.0213	0.0598	3.19
0.0553	0.0058	0.0601	0.0438	0.0386	0.0341	0.0681	0.0255	0.0717	8.61
0.0564	0.0044	0.0822	0.0453	0.0239	0.0368	0.069	0.0251	0.0722	9.58
0.0403	0.0031	0.0503	0.0327	0.036	0.0288	0.0437	0.0173	0.0486	7.64
0.0519	0.0054	0.063	0.0411	0.0362	0.0321	0.0639	0.0239	0.0673	8.52

0.0547	0.0042	0.0651	0.0429	0.0399	0.0343	0.0628	0.0256	0.0717	3.19
0.0452	0.0088	0.0547	0.0378	0.0238	0.0284	0.0478	0.0193	0.0538	3.29
0.0547	0.0052	0.0379	0.0732	0.0206	0.0295	0.0634	0.0253	0.0628	3.51
0.0471	0.0057	0.0568	0.0378	0.0311	0.0295	0.0542	0.0214	0.0599	
0.0526	0.0049	0.0649	0.0416	0.0289	0.0333	0.0639	0.0234	0.0684	
0.0536	0.0044	0.0725	0.0427	0.0226	0.0348	0.065	0.0235	0.0693	
0.0551	0.0047	0.0689	0.0435	0.0231	0.0355	0.0662	0.0238	0.0718	
0.0435	0.0038	0.0559	0.0345	0.0333	0.0282	0.0505	0.0198	0.0556	
0.0545	0.0047	0.0636	0.0432	0.0252	0.0349	0.0659	0.0239	0.0707	
0.0524	0.0041	0.0764	0.0421	0.0222	0.0342	0.0642	0.0233	0.0671	
0.0632	0.0053	0.0789	0.0498	0.0264	0.0407	0.0758	0.0273	0.0823	
0.0601	0.0047	0.0875	0.0483	0.0255	0.0392	0.0735	0.0267	0.0769	
0.0429	0.0033	0.0536	0.0347	0.0384	0.0307	0.0466	0.0184	0.0517	
0.0593	0.005	0.0741	0.0468	0.0248	0.0382	0.0712	0.0256	0.0773	
0.0583	0.0045	0.0623	0.0457	0.0425	0.0365	0.0669	0.0273	0.0763	
0.0481	0.0094	0.0583	0.0403	0.0254	0.0303	0.0509	0.0206	0.0573	
0.0583	0.0055	0.0404	0.078	0.0219	0.0314	0.0675	0.0269	0.0669	
0.0519	0.0048	0.0629	0.0403	0.0241	0.0323	0.062	0.0227	0.0663	
