# Supplementary material for: Agronomic Trait Variations and Ploidy Differentiation of Kiwiberries in Northwest China: Implication for Breeding
Source: Front Plant Sci. 2017 May 11;8:711. doi: 10.3389/fpls.2017.00711 (PMC5426280; doi:10.3389/fpls.2017.00711)
Supplement: Supplementary file 2 [file DataSheet2.docx]

**SUPPLEMENTARY DATA**

Fig. S1. Morphological variations in the leaves of (A) *A. arguta* var. *arguta*, (B) *A. melanandra*, (C) octaploid and (D, F) decaploid *A. arguta* var. *giraldii*, and (E) the intermediate phenotypes of *A. arguta* var. *arguta* and *A*. *melanandra*, observing using a microscope.


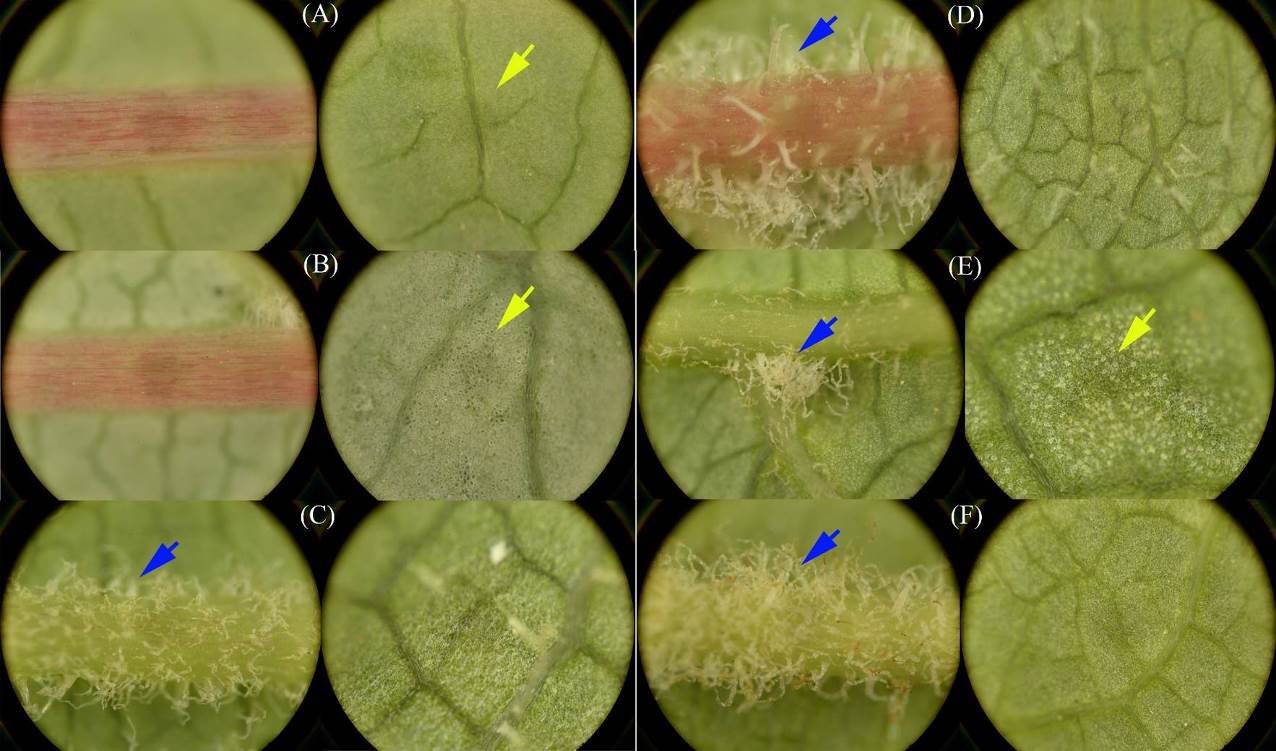


Fig. S2. The flower morphology of *A. arguta* var. *arguta*, *A. arguta* var. *giraldii* and *A. melanandra*.


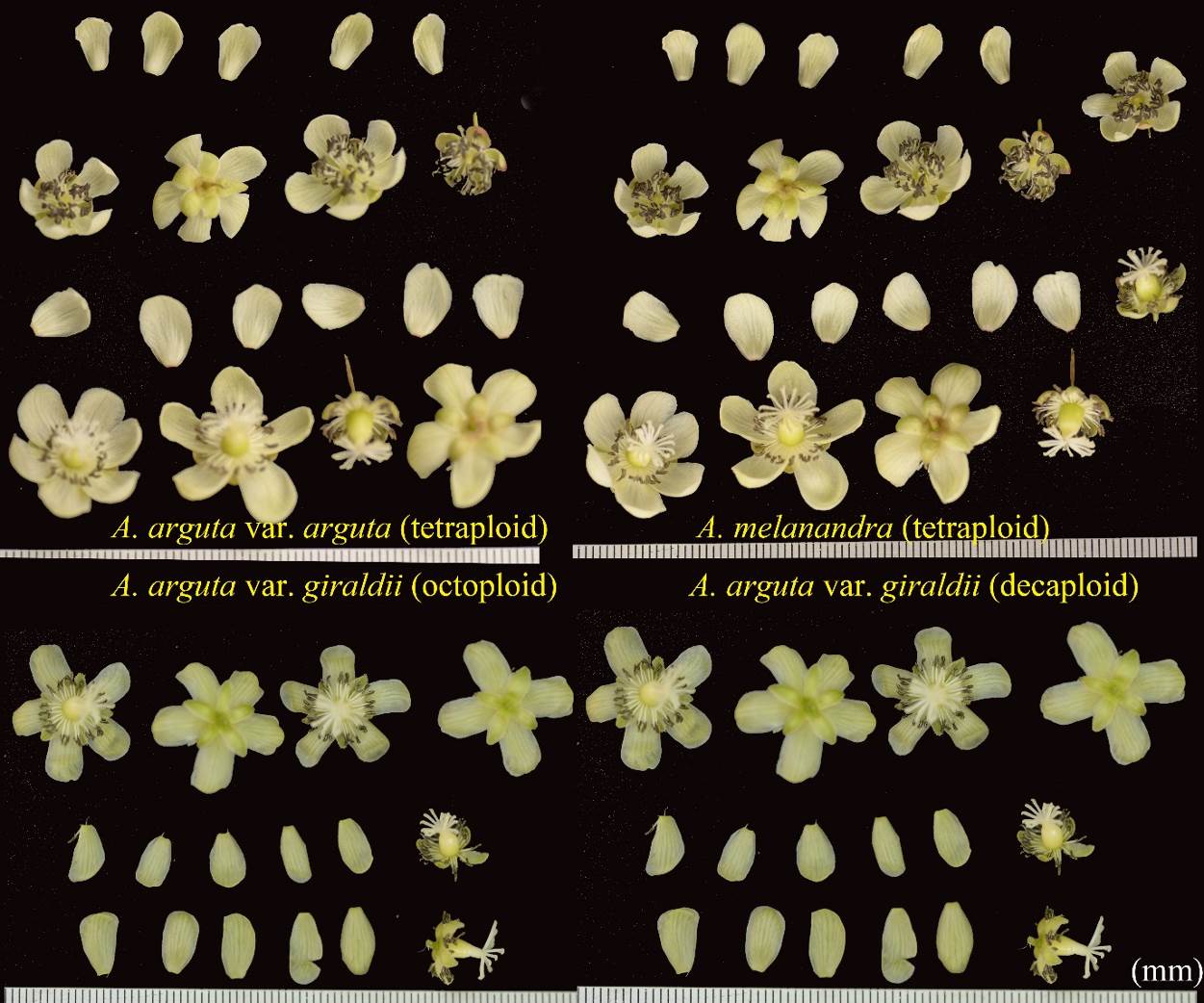


Fig. S3. The flowering time divergence of ploidy races in *A. arguta* var. *arguta*, *A. arguta* var. *giraldii* and *A. melanandra*.

**
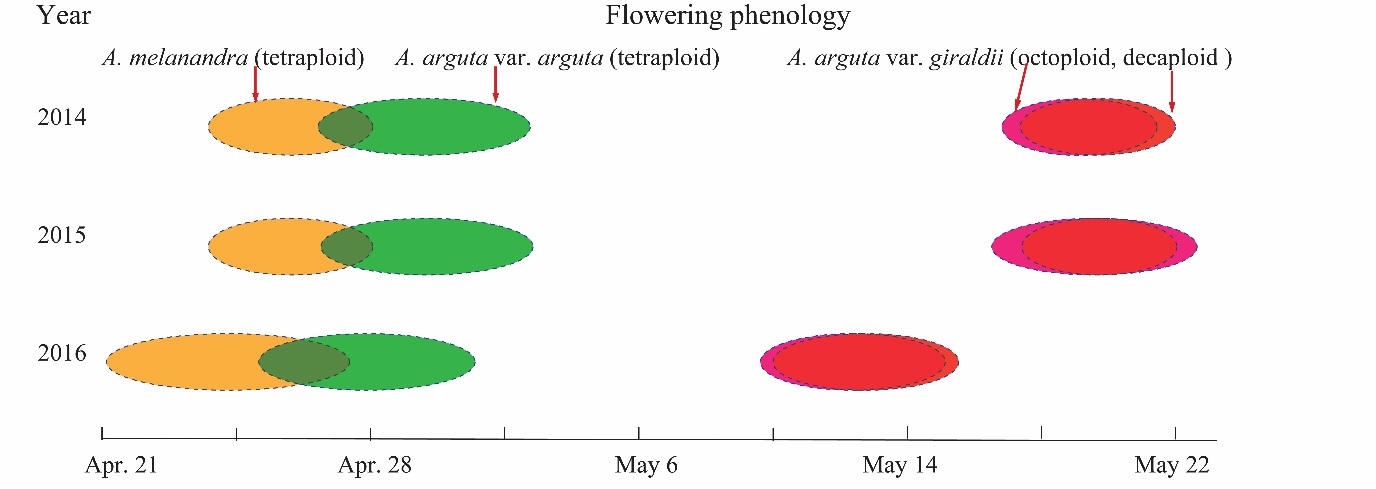
**

Table S3. Pearson correlation coefficients between ploidy levels and climate data of *A. arguta* var. *arguta*, *A. arguta* var. *giraldii* and *A. melanandra*. in Qinling Mountain.

|  |  | Altitude (m a.s.l) | Annual cumulative sunshine hours (hour) | Monthly mean maximum temperature(℃) | Monthly mean minimum temperature(℃) | Extreme maximum temperature (℃) | Extreme minimum temperature(℃) | Monthly mean minimum humidity (%) | Monthly mean maximum humidity (%) | Monthly mean minimum precipitation(mm) | Monthly mean maximum precipitation(mm) |
| --- | --- | --- | --- | --- | --- | --- | --- | --- | --- | --- | --- |
| Ploidy levels | Pearson's correlation coefficients | 0.232^*^ | -0.222^*^ | -0.072 | 0.029 | -0.125 | -0.013 | 0.170 | 0.092 | -0.228^*^ | 0.034 |
|  | *P*-value | 0.011 | 0.015 | 0.435 | 0.751 | 0.174 | 0.886 | 0.064 | 0.317 | 0.013 | 0.717 |

**Significant at *p* ≤ 0.01, *Significant at *p* ≤ 0.05.

Table S4 Pearson correlation coefficients between ploidy levels and fruit and leaf characters in *A. arguta* var. *arguta*, *A. arguta* var. *giraldii* and *A. melanandra*.

|  |  | Leaf length | Leaf width | Petiole length | Flower diameter | Fruit length | Fruit greater diameter | Fruit Lesser diameter | Fruit weight (g) | Soluble Solids Content | Ascorbic Acid | Total sugar | Total acid | Total amino acid | Asparagine | Threonine | Serine |
| --- | --- | --- | --- | --- | --- | --- | --- | --- | --- | --- | --- | --- | --- | --- | --- | --- | --- |
| Ploidy levels | Pearson's correlation coefficients | 0.424** | 0.478** | 0.362** | 0.191* | 0.113 | -0.671** | -0.652** | -0.531** | -0.197* | 0.434** | -0.526** | -0.390** | 0.794** | 0.710** | 0.728** | 0.575** |
|  | Ploidy levels | 0.000 | 0.000 | 0.000 | 0.037 | 0.222 | 0.000 | 0.000 | 0.000 | 0.032 | 0.000 | 0.000 | 0.000 | 0.000 | 0.000 | 0.000 | 0.000 |
|  |  | Glutamic acid | Proline | Glycine | Alanine | Cystine | Valine | Methionine | Isoleucine | Leucine | Tyrosine | Phenylalanine | Lysine | Histidine | Arginine | Total Anthocyanin |  |
| Ploidy levels | Pearson's correlation coefficients | 0.647** | 0.603** | 0.696** | 0.622** | 0.540** | 0.690** | 0.463** | 0.530** | 0.613** | 0.539** | 0.684** | 0.1782743 | 0.607** | 0.665** | 0.395** |  |
|  | Ploidy levels | 0.000 | 0.000 | 0.000 | 0.000 | 0.000 | 0.000 | 0.000 | 0.000 | 0.000 | 0.000 | 0.000 | 0.052 | 0.000 | 0.000 | 0.004 |  |

**Significant at *p* ≤ 0.01, *Significant at *p* ≤ 0.05.
